# Decision models of emission reduction considering CSR under reward-penalty policy

**DOI:** 10.1371/journal.pone.0285895

**Published:** 2023-07-11

**Authors:** Yang Wang, Xiuling Chen, Xideng Zhou

**Affiliations:** 1 School of Business Administration, Jiangxi University of Finance and Economics, Nanchang, China; 2 School of Business, Minnan Normal University, Zhangzhou, China; 3 Business School, Wuyi University, Nanping, Fujian, China; 4 School of Economics and Management, Yuzhang Normal University, Nanchang, China; University of Murcia: Universidad de Murcia, SPAIN

## Abstract

For the two emission reduction technologies of clean process (CT Mode) and end-of-pipe pollution control technology (ET Mode), this paper constructs production and low-carbon R&D decision-making models considering consumers’ green preference, and discusses the impact of social responsibility on firm’s decision-making, profit and social welfare. Then, the difference of optimal decision, profit and social welfare is analyzed when the firm adopt two emission reduction technologies with or without reward-penalty policy. The main conclusions of this paper are as follows: (1) Whether using clean process technology or end-of-pipe pollution control technology, consumers’ green preference behavior can increase corporate profit. When consumers’ green preference is small, consumers’ green preference is negatively correlated with social welfare. When consumers’ green preference is large, consumers’ green preference is positively correlated with social welfare. (2) Corporate social responsibility is conducive to improving the level of social welfare, not conducive to the increase of corporate profits. (3) When the reward and punishment intensity is small, the reward-penalty policy cannot effectively motivate the firm to assume social responsibility. Only when the reward and punishment reaches a certain level, the mechanism can have an incentive effect on the firm, and the government can actively implement the mechanism. (4) When the market scale is small, the adoption of end-of-pipe pollution control technology is more beneficial to the firm; When the market scale is large, it is beneficial for the firm to adopt clean technology. (5) If the efficiency of end-of-pipe pollution control and emission reduction is much higher than that of clean process, the firm should choose end-of-pipe pollution control technology, otherwise choose clean process.

## 1. Introduction

The development of industry and the consumption of fossil energy have promoted the improvement of living standards, but also brought many environmental problems such as greenhouse effect, extreme climate, air pollution, water pollution, etc. Green development has become a global consensus. In order to alleviate environmental pressure, many countries and regions have formulated specific carbon emission reduction targets, constantly strengthened and improved relevant legislation, and exerted influence on manufacturers through various administrative or economic policies, to guide manufacturers to develop and introduce low-carbon and energy-saving products and achieve sustainable development of manufacturers. As early as the beginning of 1970, President Nixon signed the National Environmental Policy Act (NEPA) passed by Congress. After Finland and Sweden introduced carbon dioxide emission taxes in the 1990s, Ireland, Denmark, Norway and Switzerland have also successively implemented carbon dioxide emission taxes. In 2005, the EU imposed carbon emission limits on enterprises and allowed them to trade carbon quotas. In 2021, the House of Representatives of the United States passed President Biden’s $1.75 trillion Build Back Better Act. The bill includes 555 billion US dollars to deal with climate change. It will vigorously promote the development of wind power, photovoltaic, energy storage, new energy vehicles and other industries by extending tax relief and increasing tax credits. In order to reduce dependence on fossil energy and improve environmental quality, some countries have introduced the global ban on fuel vehicles. For example, Germany and the United Kingdom have implemented the ban on fuel vehicles in 2030 and 2040 respectively.

Through the above analysis, it is imperative to develop a "low-carbon economy" characterized by low energy consumption and low pollution. With the increasing awareness of low carbon, consumers are increasingly concerned about the low carbon properties of products. For example, from 2011 to 2015, the compound growth of consumers who bought green products at higher prices on Alibaba’s platform was more than 80%; In 2017, green consumers on the JD platform who pay attention to the ecological environment increased by 62% year on year. According to a research report released by the European Commission in 2014, 75% of respondents expressed their willingness to pay higher prices for environmental protection products, compared with 72% in 2011. This shows that the proportion of consumers willing to accept environmentally friendly products with higher prices is increasing. Under the low carbon awareness of consumers, enterprises can not only seize the market opportunity, but also improve their social image by taking low carbon measures. The Stern Report points out that the global market for low-carbon products is expected to reach 500 billion US dollars by 2050 [1].

For manufacturers, low-carbon economy and sustainable development include two important aspects. (1)Manufacturers are required to assume social responsibility, actively respond to the demands of stakeholders, and promote sustainable and harmonious development of economy, society and environment through transformation and innovation. Corporate social responsibility means that when an enterprise gains profits through production and operation, it is responsible not only for shareholders and employees, but also for consumers, the environment and society. Therefore, organizations undertaking corporate social responsibility should not only consider maximizing their own economic benefits, but also pay attention to human values in the production process, and pay more attention to their contributions to the environment, consumers and society. (2)Enterprises are required to save energy and reduce emissions. The most fundamental way to save energy and reduce emissions is to rely on manufacturers’ independent technology innovation of emission reduction. Emission reduction technologies include cleaner process and end-of-pipe pollution control technologies, called CT Mode and ET Mode respectively. In fact, the mechanism of cleaner process and end treatment technology innovation on manufacturers’ pollution control is different. The clean process uses advanced production processes and equipment, including the use of substitutes for toxic and harmful raw materials, to achieve the recycling of waste, so as to improve the utilization rate of raw materials and energy and the profitability of products, so that manufacturers do not produce or produce as little waste as possible in the production process. For the pollutants generated at the end of the production process, the end-of-pipe pollution control technologies shall implement effective treatment technology to reduce the emission of pollutants.

Then, what are the differences in emission reduction performance of enterprises under different pollution control technologies?

What kind of pollution control technology should enterprises choose?

What factors affect their choice?

It can be seen that in the process of green transformation, how to choose emission reduction technologies to gain market competitive advantage has become one of the issues that need to be concerned.

In addition, in order to alleviate environmental pressure, how should the government formulate corresponding environmental policies to encourage enterprises to reduce emissions and research and development, encourage enterprises to assume social responsibility, so as to make the economy and environment develop in a coordinated manner?

The answers to the above questions will be of great significance to building an environment-friendly society and sustainable economic development.

The main contributions of this work are as follows. First, according to the working principles of clean technology and end-of-pipe pollution control, this paper improves the corresponding models in reference [38] and constructs emission reduction modes under different technology pollution control modes. Second, for different emission reduction modes, the government reward-penalty policy is constructed. In the past, most of the literature only considered punitive measures, such as tax collection, or incentive measures, such as subsidies, etc. Few literature combined incentives and punitive measures to study firm emission reduction. Thirdly, we analyze the impact of corporate social responsibility on firm decision-makings and social welfare, and provide one of the feasibility for the government to formulate reward-penalty policy. In addition, we provide some managerial implications for sustainable development considering energy-saving investment and behavioral concerns.

## 2. Literature review

This paper is chiefly related to three streams of literature. The first stream is the research on corporate social responsibility. In the real business environment, enterprises are not only important economic subjects, but also social participants, closely linked with many stakeholders. Corporate social responsibility (CSR) is a kind of corporate behavior in which an enterprise demonstrates its social and moral responsibilities to its stakeholders, which determines that an enterprise can realize its social value by implementing its social responsibilities to its stakeholders [2]. In addition, some empirical analysis also shows that enterprises with social responsibility have more competitive advantages than enterprises only considering economic profits [3, 4]. Can enterprises improve the efficiency of government subsidies by implementing CSR, so as to achieve their own optimal benefits? At this stage, scholars’ research on CSR mainly focuses on the impact of CSR on supply chain decision-making and coordination. Panda et al. [5] used Nash bargaining to coordinate the supply chain, and also rationally allocated consumer surplus. Liu et al. [4] introduced the government subsidy mechanism, discussed the impact of government subsidies on the profits of supply chain members, corporate social responsibility efforts and social welfare, and determined the relationship between the optimal government subsidy rate and corporate social responsibility level. Zhu and Lai [6] studied corporate social responsibility from the perspective of suppliers, and provided a new theoretical perspective for multinational companies to cultivate relationships with suppliers in emerging countries through hierarchical regression analysis. Shib [7] dealed with a newsvendor inventory model in light of green product marketing of corporate social responsible firms. Swami et al. [8] pointed out the expectations of stakeholders on enterprises and supply chains. In particular, for those enterprises that increasingly want to incorporate corporate social responsibility into supply chain decision-making, corporate social responsibility has evolved from a voluntary initiative to a strategic decision, which is consistent with the views of stakeholders in Freeman [9], which describes corporate social responsibility as the degree of concern for stakeholders. Yao et al. [10] discussed the pricing and sales effort decision of corporate social responsibility on closed-loop supply chain in the context of closed-loop supply chain. Song et al. [11] constructed a two-stage closed-loop supply chain, studied recycling strategy of closed-Loop supply chain considering CSR under the government’s reward-penalty policy. Wu et al. [12] explored the impacts of different corporate social responsibility (CSR)-undertaking-mode choices on the carbon-emission reduction and technological innovation decisions of the low-carbon supply chain. With respect to the coordination problems of closed-loop supply chains led by retailers, Zhao et al. [13] established some models considering manufacturer’s CSR, exploited them to compare the optimal decisions under centralized and decentralized decisions, explored the impact of CSR on supply-chain decisions, and designed a coordination mechanism through two-step pricing. Wang et al. [14] considered a three-echelon closed-loop supply chain consisting of a manufacturer with corporate social responsibility (CSR), a retailer with sales effort, and a third party who is responsible for collecting used products. Zhang and Li [15] put CSR into account in a dual-channel closed-loop supply chain, constructed two models for the retailer to implement or not implement CSR activities, and analyzed the decisions obtained under optimal solutions. Considering the product pricing, CSR level, and service level in the supply chain, Xin et al. [16] employed the Stackelberg game to depict supply chain participants’ optimal decisions and analyzed the influence of explanatory variables on the optimal decision with retailer’s payment methods. Yang et al. [17] considered a three-tier supply chain system and used stochastic differential game to study the CSR coordination of the supply chain.

The second stream is the research on government’s environmental protection policy. The emission reduction has become an inevitable trend. The government has been acting as an important role in the operation and management of supply chain. Wang et al. [18] examined governmental carbon tax policies and their relationships with supply chains. Xu et al. [19] considered four different governmental subsidy strategies including NS Scenario, MS Scenario, RS Scenario, SS Scenario. It can be concluded that subsidizing to both members is more profitable for supply chain members and government in terms of environment protection and economic development. Lyu et al. [20] investigated how government regulations (cap-and-trade, strict carbon cap and carbon tax) to curb emissions differs systematically, and the manufacturer’s recycling decision. Yu et al. [21] investigated the carbon emission reduction efforts and pricing decisions for a two chains system under carbon taxation, found that as long as carbon taxation occurs, each supply chain members’ vertical collaboration wound drop carbon emission rate and product price. Duan et al. [22] analyzed the impact of the government’s carbon trading price (CTP), carbon tax (CT) and low-carbon product subsidy (LPS) policies on the supply chain network equilibrium. Cao et al. [23] developed theoretical models considering retailer channel selections, different power structures, and cap-and-trade policy. They found that the optimal channel selection of the retailer is affected by the annual service fee, the power structure and the carbon trading price. Considering consumer’s low carbon preference and carbon tax under the cap-and-trade regulation, Cheng et al. [24] investigated the optimal strategies for an economic constrained closed-loop supply chain network. Yang and Xu [25] considered the competition among different manufacturers and multiple demand markets in the supply chain network, then examined and compared their equilibrium decision conditions under three common carbon policies: carbon cap, carbon cap-and-trade, and carbon tax.

As for the research on the government’s reward-penalty policy, most of the literatures take the closed-loop supply chain as the research object to study the impact of the reward-penalty policy on the enterprise recovery decision and social welfare. Niu et al. [26] studied the government punishment and subsidy in the process of sustainable fashion procurement, and believed that the goal of both pursuing sustainability and maximizing social welfare is contradictory, and it is necessary to find a balance between government punishment and subsidy. Zhang et al. [27] discussed the government reward-penalty mechanism (RPM) between two competing manufacturers and a recycler in closed-loop supply chain (CLSC) under asymmetric information. Chen et al. [28]studied the manufacturer’s investment choice of emission reduction technology under the background of carbon quota trading policy. Chen et al. [29] studied the green supply chain led by a manufacturer and followed by a retailer. Using Stackelberg game theory, they built game models with no reward-penalty policy and with reward-penalty policy respectively. Through comparative analysis, they found that the government’s reward-penalty policy can not only improve the level of product energy saving, but also improve the level of social welfare. By establishing an evolutionary game model, Zhang et al. [30] focused on analyzing how paper-making enterprises choose their own emission reduction strategies under the reward-penalty policy. It further analyzed how social welfare changes under the reward-penalty policy.

The third stream of related literature concerns research on enterprise’s emission reduction strategy. In the face of environmental deterioration and the increasing awareness of environmental protection of consumers, enterprises will have to take positive measures to prevent environmental pollution in order to achieve sustainable development. Liu et al. [31] focused on how the producer inspires his cooperative research partner to reduce carbon emission, by developing a menu of incentive contracts both in research and development (R&D) stage and recycling stage. Chen et al. [28] explored firms’ green R&D cooperation behavior in a two-echelon supply chain in which a manufacturer and a retailer first cooperate to invest green R&D and then organize production according to a wholesale price contract. Main findings show that the R&D cooperation’s improvement of firms’ economic performance is mainly determined by firms’ own green contribution level. Zhang et al. [32] studied a supply chain adopting joint emission reduction strategy, investigated under what circumstance the environmental regulation can effectively result in higher emission reduction efforts. Ma et al. [33] considered a supply chain system composed of one manufacturer and one retailer, where the manufacturer invests in green emission reduction technology (GERT) to reduce carbon emissions, and the retailer invests in information disclosure technology to transmit the higher greenness quality of products to consumers. Liu et al. [34] explored the impacts of supply chain competition on manufacturers’ CDMs (clean development mechanisms) introduction strategies, showed that in the OT model (a model with a monopoly manufacturer and two competing retailers), it is optimal for the manufacturer to introduce a CDMs. Che et al. [35] constructed a dual-channel supply chain decision-making model composed of low-carbon emission reduction manufacturers and retailers and studied the optimal decision-making problem based on emission reduction R&D and per unit product emission reduction. Guo et al. [36] established models for multiple recycling structures to identify the optimal emission reduction strategy of manufacturers. Li et al. [37] studied government participation in supply chain low-carbon technology R&D. It was found that the development of a mechanism for horizontal technology R&D among enterprises can reduce the financial pressure on the government to implement compensation strategies and improve the effectiveness and performance of supply chain emission reduction. Cheng and Zhang [38] studied the problem of emission reduction technology selection in a green supply chain composed of a single manufacturer and a single retailer through game theory, and further discussed the impact of penalty sharing contract on green supply chain decision-making. Hanieh et al. [39] studied financing a sustainable supply chain through green bonds.

To sum up, previous scholars have done a lot of research on corporate social responsibility, government environmental policies, and corporate emission reduction strategies. It is mainly reflected in the following aspects: at this stage, (1) previous literature mainly focused on the impact of corporate social responsibility on supply chain decision-making and coordination, inventory decision-making, and government subsidy efficiency, (2) analyzed how carbon trading prices, carbon quotas, carbon taxes, subsidies, and reward-penalty policy affect supply chain decision-making and performance, and (3) discussed the role of enterprise emission reduction cooperation and competition on enterprise economic performance.

However, in spite of researcher’s effort and with respect of their searches, some gaps were observed in previous researches that was tried to addressed them in this research. So, according to the literature review, in terms of enterprise emission reduction technology, relevant research rarely divides emission reduction technology into clean process and end-of-pip pollution control technology. However, different pollution control technologies have different effects on enterprises. Therefore, under different technological pollution control models, the profit function of enterprises will also have essential differences. Although literature [38] has conducted a simple study on this issue, it only considers economic responsibility, but ignores the social responsibility of enterprises. So what impact will corporate social responsibility have on corporate emission reduction behavior, performance and social welfare? What are the differences in emission reduction behavior, performance and social welfare of enterprises under different pollution control modes? Can the government’s reward-penalty policy motivate enterprises to reduce emissions? How do enterprises choose technology pollution control mode? These problems have not been studied in the existing literature. In order to fill the research gap, (1) we build a corporate decision-making model under the clean process and end-of-pipe pollution control technology modes, analyze the changes of corporate profits and social welfare before and after assuming social responsibility, and compare the differences of corporate emission reduction behavior, performance and social welfare under different pollution control modes. (2) By comparing the difference between profit and social welfare with or without government reward-penalty policy, the scope of application of this mechanism is proposed. (3) In the proposed model, we build a demand function including consumer’s green preference, which can better reflect the actual situation.

The structure of this paper is as follows: The second part mainly reviews the literature from three aspects: corporate social responsibility, reward-penalty policy and low-carbon emission reduction strategy. The third section puts forward basic assumptions and describes the research issues. Section 4–5 is the core content of this paper, which constructs the enterprise emission reduction decision-making model with or without reward-penalty policy and corporate social responsibility. The sixth part is the conclusion of this paper.

## 3. Model development

This paper takes a manufacturer that discharges pollutants as the research object. The government encourages the manufacturer to reduce pollutant emissions through the reward-penalty policy. The manufacturer can choose one of two technical modes to reduce pollutant emissions, namely, cleaner process pollution control technology mode (called CT Mode) and end-of-pipe pollution control technology mode (called ET Mode). This paper studies manufacturer’s decision-makings with and without government reward-penalty policy, which provides theoretical support for enterprises to make correct decisions, and analyzes the impact of relevant parameters on profits, which provides a basis for enterprises to choose emission reduction technology models; It also analyzes the impact of consumers’ green preference, corporate social responsibility and reward-penalty policy on corporate profits and social welfare, which provides reference for the government to formulate effective policies.

**Hypothesis 1:** In this paper, it is assumed that the manufacturer produces a unit of product and emits *m* unit of pollutant, and the production cost of a unit of product is *c*. The R&D costs of CT Mode and ET Mode are $\frac{1}{2}\eta_{c}k_{c}^{2}$ and $\frac{1}{2}\eta_{e}k_{e}^{2}$ respectively, where *η* is the R&D efficiency, *k*_*c*_ and *k*_*e*_ are the R&D level. The relationship between pollutant discharge per unit product and R&D level under CT Mode is *e* = *m*‒*l*_*c*_*k*_*c*_, which indicates that the pollution control technology reduces the intensity of pollution discharge process and belongs to process pollution control; The relationship between pollutant discharge per unit product and R&D level under ET Mode is $e=m-\frac{l_{e}k_{e}}{q}$, which indicates that the technology does not interfere with the pollutant discharge process. Where *q* is the yield.

**Hypothesis 2:** In order to improve the environmental quality, the government formulates a reward-penalty policy *βq*(*e*_0_‒*e*) for pollutant discharging enterprises, that is, when the pollutants *e* of enterprises are lower than the standard *e*_0_ set by the government, the enterprises will be rewarded as *βq*(*e*_0_‒*e*), otherwise they will be punished, where *β* > 0 is the government’s reward and punishment for the manufacturer’s unit emission reduction level.

**Hypothesis 3:** Without considering the retail link, manufacturers directly sell products to consumers. The market demand function is *q* = *a* ‒ *bp* ‒ *γe*, where *a* is the market capacity, *P* is the unit product price, *b* is the price sensitivity, *γ* is the degree of consumers’ aversion to pollutants, and the reverse demand function is $p=\frac{a}{b}-\frac{1}{b}q-\frac{1}{b}\gamma e$. Let $\theta =\frac{1}{b}$, and rewrite it as *p* = *aθ*‒*θq*‒*θγe*.

**Hypothesis 4:** Consumer surplus is an important indicator to weigh consumer welfare. According to the definition of Panda et al. [2], consumer surplus is the difference between the highest price consumers are willing to pay for a certain amount of goods and the actual market price of the goods, which can be expressed as:

$$CS=\int_{p}^{p_{\max}}Qdp=\frac{q^{2}}{2}$$ (1)

Where, *p*_max_ represents the price when the demand is 0.

**Hypothesis 5:** Based on stakeholder theory [9], corporate social responsibility can be seen as a series of activities to improve the welfare of its stakeholders, aiming to enhance social benefits. The environmental benefits of the manufacturer under social responsibility can be expressed as:

$$\pi^{E}=tCS=t\frac{q^{2}}{2}$$ (2)

Where, *t* is the level of social responsibility undertaken by manufacturers, 0≤*t*≤1, which indicates the sensitivity of environmental benefits obtained by manufacturers to consumer surplus. *t* = 0 indicates that the manufacturer pursues the maximization of economic benefits; *t* = 1 means that the manufacturer pursues the maximization of overall benefits (economic benefits and environmental benefits).

**Hypothesis 6:** The pollutant discharge will cause damage to the environment. With reference to the research of Cheng et al. [29], the damage function under the CT Mode is *T* = *veq* the damage function under the ET Mode is $T=vq\frac{e^{2}}{2}$, where *v*(*v* > 0) is the pollution damage coefficient. The assumption of pollution damage function shows that there are differences in the harmful degree of pollution emissions under different pollution control modes. In the process of pollution control, the CT Mode can filter out toxic substances that cause fatal harm to the environment, thereby reducing environmental damage; However, the ET Mode does not interfere with the pollutant discharge process, and the damage caused is actually greater than the CT Mode.

## 4. No reward-penalty policy

### 4.1 No corporate social responsibility without reward-penalty policy (NN case)

The manufacturer does not assume social responsibility, that is, the situation where the manufacturer lacks social responsibility. In the pharmaceutical manufacturing industry, maintaining a sense of social responsibility will bring corresponding costs, so this benchmark situation generally exists in the early pharmaceutical manufacturing industry and individual pharmaceutical manufacturers at this stage. In this case, the manufacturer’s income is purely economic.

#### 4.1.1 Cleaner process pollution control technology (called CT Mode)

When the manufacturer adopts CT Mode, the manufacturer’s decision objective function is

$$\pi_{c}^{NN}=(a\theta -\theta q-\theta \gamma e)q-cq-\frac{1}{2}\eta_{c}k_{c}^{2}$$ (3)

In the right part of Formula (3), the first item represents the total revenue of the manufacturer from selling green products, the second item represents the manufacturer’s production cost, and the third item represents the manufacturer’s research and development cost.

According to the solution idea in literature [40–43], differentiating $\pi_{c}^{NN}$ with respect to yield ($q_{c}^{NN*}$) and R&D level ($k_{c}^{NN*}$), we have

$$\frac{d\pi_{c}^{NN}}{dq_{c}^{NN}}=(a\theta -\theta q-\theta \gamma e)-\theta q-c,$$ (4)

$$\frac{d\pi_{c}^{NN}}{dk_{c}^{NN}}=\theta \gamma l_{c}q-\eta_{c}k_{c}^{NN},$$ (5)

$$\frac{d^{2}\pi_{c}^{NN}}{dq_{c}^{NN}dk_{c}^{NN}}=\frac{d^{2}\pi_{c}^{NN}}{dk_{c}^{NN}dq_{c}^{NN}}=\theta \gamma l_{c},$$ (6)

$$\frac{d^{2}\pi_{c}^{NN}}{dq_{c}^{NN}{}^{2}}=-2\theta ,$$ (7)

$$\frac{d^{2}\pi_{c}^{NN}}{dk_{c}^{NN}{}^{2}}=-\eta_{c}.$$ (8)

Now, the hessian matrix at ($q_{c}^{NN*}$, $k_{c}^{NN*}$) is

$$M=\left[\begin{array}{cc} \frac{d^{2}\pi_{c}^{NN}}{dq_{c}^{NN}{}^{2}} & \frac{d^{2}\pi_{c}^{NN}}{dk_{c}^{NN}dq_{c}^{NN}}\\ \frac{d^{2}\pi_{c}^{NN}}{dk_{c}^{NN}dq_{c}^{NN}} & \frac{d^{2}\pi_{c}^{NN}}{dk_{c}^{NN}{}^{2}} \end{array}\right]=\left[\begin{array}{cc} -2\theta & \theta \gamma l_{c}\\ \theta \gamma l_{c} & -\eta_{c} \end{array}\right]$$

**Proposition 1:** When the manufacturer adopts CT Mode under NN case, the profit function of the manufacturer $\pi_{c}^{NN}$ attains maximum at ($q_{c}^{NN*}$, $k_{c}^{NN*}$) if *η*_*c*_(2*θ*) > (*θγl*_*c*_)^2^ and *aθ* ‒ *θγm*>*c* hold. The optimal R&D level and yield under CT Mode are shown below.

$$q_{c}^{NN*}=\frac{\eta_{c}(a\theta -\theta \gamma m-c)}{\eta_{c}(2\theta )-{\left(\theta \gamma \right)}^{2}l_{c}{}^{2}},$$ (9)

$$k_{c}^{NN*}=\frac{\theta \gamma l_{c}(a\theta -c-\theta \gamma m)}{\eta_{c}(2\theta )-{\left(\theta \gamma \right)}^{2}l_{c}{}^{2}}.$$ (10)

**Proof:** Now for maximum value of $\pi_{c}^{NN}$, $\frac{d\pi_{c}^{NN}}{dq_{c}^{NN}}=0=\frac{d\pi_{c}^{NN}}{dk_{c}^{NN}}$ . $\frac{d\pi_{c}^{NN}}{dq_{c}^{NN}}=0$$\overset{yields}{\to }$$q_{c}^{NN*}$ $=\frac{\eta_{c}(a\theta -\theta \gamma m-c)}{\eta_{c}(2\theta )-{\left(\theta \gamma \right)}^{2}l_{c}{}^{2}}$ and $\frac{d\pi_{c}^{NN}}{dk_{c}^{NN}}=0$$\overset{yields}{\to }$$k_{c}^{NN*}$$=\frac{\theta \gamma l_{c}(a\theta -c-\theta \gamma m)}{\eta_{c}(2\theta )-{\left(\theta \gamma \right)}^{2}l_{c}{}^{2}}$. Now at ($q_{c}^{NN*}$, $k_{c}^{NN*}$), $\frac{d^{2}\pi_{c}^{NN}}{dqdk_{c}}=\frac{d^{2}\pi_{c}^{NN}}{dk_{c}dq}=\theta \gamma l_{c}$ and $\frac{d^{2}\pi_{c}^{NN}}{dq^{2}}=-2\theta <0$ and $\frac{d^{2}\pi_{c}^{NN}}{dk_{c}{}^{2}}=-\eta_{c}<0$. The determinant of hessian matrix *M* is $\frac{d^{2}\pi_{c}^{NN}}{dq^{2}}\frac{d^{2}\pi_{c}^{NN}}{dk_{c}{}^{2}}-{\left(\frac{d^{2}\pi_{c}^{NN}}{dqdk_{c}}\right)}^{2}=2\theta \eta_{c}-{\left(\theta \gamma l_{c}\right)}^{2}$. If *η*_*c*_(2θ) > (*θγl*_*c*_)^2^, the hessian matrix *M* at ($q_{c}^{NN*}$, $k_{c}^{NN*}$) is negative definite. Therefore, objective function $\pi_{c}^{NN}$ attains maximum at ($q_{c}^{NN*}$, $k_{c}^{NN*}$). Since the feasibility of the Eqs (9–10) require positive values of $q_{c}^{NN*}$ and $k_{c}^{NN*}$, *aθ* ‒ *θγm*>*c* must hold.

Substituting Eqs (9) and (10) into (3), we can get the manufacturer’s profit as

$$\pi_{c}^{NN*}=[a\theta -c-\theta \gamma (m-l_{c}k_{c}^{NN}{}^{*})]q_{c}^{NN}{}^{*}-\theta q_{c}^{NN}{{}^{2}}^{*}-\frac{1}{2}\eta_{c}k_{c}^{NN2*}.$$ (11)

The social welfare function includes enterprise profit, consumer surplus, and pollution damage. The social welfare function under the clean process pollution control is:

$$F_{c}^{NN*}=\pi_{c}^{NN}+CS-vqe.$$ (12)

By substituting Eq (11) into Eq (12), we can get the social welfare level without reward-penalty policy and corporate social responsibility, as shown below:

$$F_{c}^{NN*}=\pi_{c}^{NN}-veq_{c}^{NN*}=[a\theta -c-(\theta \gamma +v)(m-l_{c}k_{c}^{NN*})]q_{c}^{NN*}-\frac{1}{2}\eta_{c}k_{c}^{NN2*}+q_{c}^{NN2*}(\frac{1}{2}-\theta )$$ (13)

#### 4.1.2 End-of-pipe pollution control technology mode (called ET Mode)

When the manufacturer adopts ET Mode, the manufacturer’s objective function is

$$\pi_{e}^{NN}=[a\theta -\theta q-\theta \gamma (m-\frac{l_{e}k_{e}}{q})]q-cq-\frac{1}{2}\eta_{e}k_{e}^{2}.$$ (14)

Taking the derivative of $\pi_{e}^{NN}$ with respect to $q_{e}^{NN}$ and $k_{e}^{NN}$, we have

$$\frac{d\pi_{e}^{NN}}{dq_{e}^{NN}}=a\theta -2\theta q-\theta \gamma m-c,$$ (15)

$$\frac{d\pi_{e}^{NN}}{dk_{e}^{NN}}=\theta \gamma l_{e}-\eta_{e}k_{e}^{},$$ (16)

$$\frac{d^{2}\pi_{e}^{NN}}{dq_{e}^{NN}dk_{e}^{NN}}=\frac{d^{2}\pi_{e}^{NN}}{dk_{e}^{NN}dq_{e}^{NN}}=0,$$ (17)

$$\frac{d^{2}\pi_{e}^{NN}}{dq_{e}^{NN2}}=-2\theta ,$$ (18)

$$\frac{d^{2}\pi_{e}^{NN}}{dk_{e}^{NN2}}=-\eta_{e}.$$ (19)

Now, the hessian matrix at ($q_{e}^{NN*}$, $k_{e}^{NN*}$) is

$$M=\left[\begin{array}{cc} \frac{d^{2}\pi_{e}^{NN}}{dq_{e}^{NN}{}^{2}} & \frac{d^{2}\pi_{e}^{NN}}{dk_{e}^{NN}dq_{e}^{NN}}\\ \frac{d^{2}\pi_{e}^{NN}}{dk_{e}^{NN}dq_{e}^{NN}} & \frac{d^{2}\pi_{e}^{NN}}{dk_{e}^{NN}{}^{2}} \end{array}\right]=\left[\begin{array}{cc} -2\theta & 0\\ 0 & -\eta_{e} \end{array}\right]$$

**Proposition 2:** When the manufacturer adopts ET Mode under NN case, the profit function of the manufacturer $\pi_{e}^{NN}$ attains maximum at ($q_{e}^{NN*}$, $k_{e}^{NN*}$) if *aθ*‒*θγm*>*c* holds. The optimal R&D level and yield under ET Mode are shown below.

$$q_{e}^{NN*}=\frac{a\theta -\theta \gamma m-c}{2\theta },$$ (20)

$$k_{e}^{NN*}=\frac{\theta \gamma l_{e}}{\eta_{e}}.$$ (21)

**Proof:** Now for maximum value of $\pi_{e}^{NN}$, $\frac{d\pi_{e}^{NN}}{dq_{e}^{NN}}=0=\frac{d\pi_{e}^{NN}}{dk_{e}^{NN}}$. $\frac{d\pi_{e}^{NN}}{dq_{e}^{NN}}=0$$\overset{yields}{\to }$$q_{e}^{NN*}$ $=\frac{a\theta -\theta \gamma m-c}{2\theta }$ and $\frac{d\pi_{e}^{NN}}{dk_{e}^{NN}}=0$$\overset{yields}{\to }$$k_{e}^{NN*}$$=\frac{\theta \gamma l_{e}}{\eta_{e}}$. Now at ($q_{e}^{NN*}$, $k_{e}^{NN*}$), $\frac{d^{2}\pi_{e}^{NN}}{dq_{e}^{NN}dk_{e}^{NN}}=\frac{d^{2}\pi_{e}^{NN}}{dk_{e}^{NN}dq_{e}^{NN}}=0$ and $\frac{d^{2}\pi_{e}^{NN}}{dq_{e}^{NN}{}^{2}}=-2\theta <0$ and $\frac{d^{2}\pi_{e}^{NN}}{dk_{e}^{NN}{}^{2}}=-\eta_{e}<0$. The determinant of hessian matrix M is $\frac{d^{2}\pi_{e}^{NN}}{dq_{e}^{NN}{}^{2}}\frac{d^{2}\pi_{e}^{NN}}{dk_{e}^{NN}{}^{2}}-{\left(\frac{d^{2}\pi_{e}^{NN}}{dq_{e}^{NN}dk_{e}^{NN}}\right)}^{2}=2\theta \eta_{e}>0$. Also, the hessian matrix M at ($q_{e}^{NN*}$, $k_{e}^{NN*}$) is negative definite. Since the feasibility of the Eq (20) requires positive value of $q_{e}^{NN*}$, *aθ* ‒ *θγm*>*c* must hold.

Substituting Eqs (20) and (21) into (14), we can get the manufacturer’s profit as

$$\pi_{e}^{NN*}=[a\theta -\theta q_{e}^{NN*}-\theta \gamma (m-\frac{l_{e}k_{e}^{NN*}}{q_{e}^{NN*}})]q_{e}^{NN*}-cq_{e}^{NN*}-\frac{1}{2}\eta_{e}k_{e}^{NN2*}.$$ (22)

The social welfare function includes enterprise profit, consumer surplus, and pollution damage. The social welfare function under the end-of-pipe pollution control technology mode is

$$F_{e}^{NN*}=\pi_{e}^{NN}+CS-vqe.$$

The optimal social welfare level under ET Mode is

$$F_{e}^{NN*}=(a\theta -c-\theta \gamma m)q_{e}^{NN*}+\theta \gamma l_{e}k_{e}^{NN*}-\frac{1}{2}\eta_{e}k_{e}^{NN*2}+(\frac{1}{2}-\theta )q_{e}^{NN*2}-vq_{e}^{NN*}\frac{{\left(m-l_{e}k_{e}^{NN*}\right)}^{2}}{2}$$ (23)

Based on the equilibrium results, the sensitivity of consumer green preference is analyzed, and inference 1 and inference 2 can be obtained.

**Inference 1:** If the manufacturer adopts CT Mode, when 2(*θl*_*c*_)^2^(*M*_1_ ‒ *η*_*c*_*θmγ*) > *η*_*c*_*θm*[*N* ‒ (*θlγ*_*c*_)^2^], the increase of consumers’ green preference will help to increase market demand $\frac{dq_{c}^{NN}}{d\gamma }>0$, increase consumer surplus $\frac{dCS_{c}^{NN}}{d\gamma }<0$, otherwise it will decrease. When (*γθl*_*c*_)^2^ *M*_2_ > 2*Nθ mγ*‒*M*_2_*N*, the increase of consumers’ green preference will help to improve the level of green R&D $\frac{dk_{c}^{NN}}{d\gamma }>0$, otherwise it will decrease. Where *N* = 2*η*_*c*_*θ*, *M*_1_ = *η*_*c*_(*aθ*‒*c*), *M*_2_ = *l_c_θ*(*aθ*‒*c*).

**Inference 2:** If manufacturers adopt ET Mode, the increase of consumers’ green preference will help to improve the level of enterprise research and development $\frac{dk_{e}^{NN}}{d\gamma }>0$, but reduce market demand $\frac{dq_{e}^{NN}}{d\gamma }<0$, and reduce consumer surplus $\frac{dCS_{e}^{NN}}{d\gamma }<0$.

This article takes a paper firm as an example. The paper industry is an important basic raw material industry closely related to the development of national economy and social undertakings. Its production is based on wood processing residues, bamboo, agricultural straw and other native plant fibers and recycled fibers such as waste paper as raw materials, and pulp is produced by chemical and mechanical methods, and then paper and paperboard are produced from pulp. The production process of pulp and paper making is long, and water, as the carrier of fiber and chemicals, runs through the whole process. The firm A has been engaged in the paper industry for many years, and has been using ET Mode to treat water pollution. With the expansion of market scale, the firm A is ready to invest in a new production line. Considering the public’s awareness of ecological and environmental protection, as well as the policy and environmental regulations, the firm A has two kinds of water pollution control technology modes to choose from, namely, CT Mode and ET Mode. According to relevant investigation, the market capacity is *a* = 10, the price sensitivity coefficient is *b =* 0.05 (*θ* = 20), the emission COD of a ton of kraft paper with a specification of 100g is *m* = 1.2, the cost is *c* = 1, and R&D efficiency are *η*_*c*_ = 0.1 and *η*_*e*_ = 0.13 respectively. The pollution coefficient of each unit of pollutant to the environment is *v* = 0.1. The emission reduction efficiency of the above two technology modes are *l*_*c*_ = 0.5 and *l*_*e*_ = 2. The customer green preference is *γ* = 0.05. Moreover, these parameter values satisfy the constraints of this paper. In the section the impacts of customer green preference parameter on optimal decisions and profits are analyzed.

**Fig 1:**
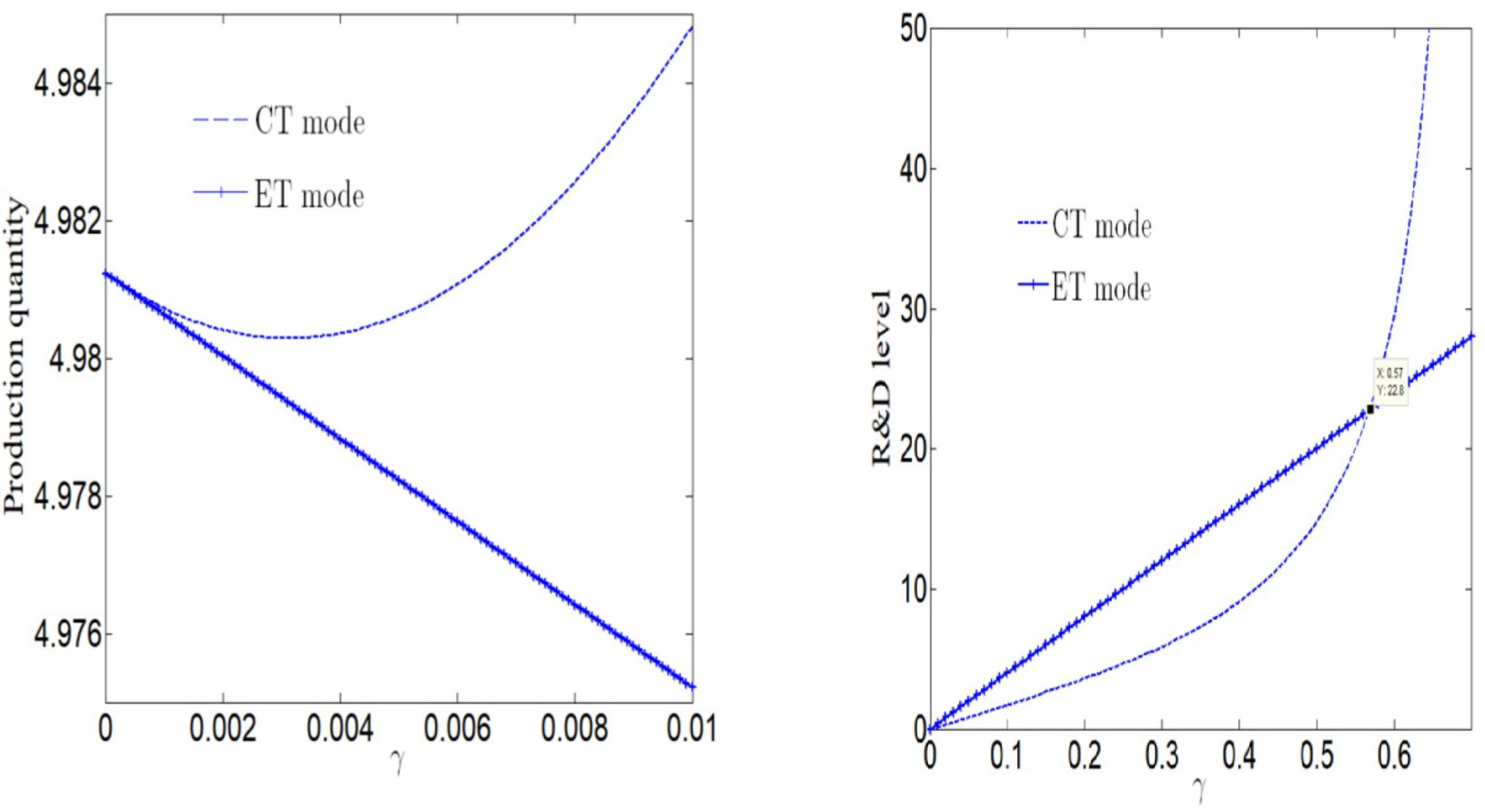
Impact of consumers’ green preference on optimal decision-making. (a) Impact of *γ* on yield. (b) Impact of *γ* on R&D level.

Fig 1(A) analyzes the impact of consumers’ green preference on the production quantity or market demand. When manufacturer adopts ET Mode, we can find that market demand decreases with the increase of consumers’ green preference. If manufacturer adopts CT Mode, the market demand will increase with the increase of consumers’ green preference. And the market demand under CT Mode is greater than that under ET Mode. Fig 1(B) shows the impact of consumers’ green preference on manufacturer’s R&D level. We found that no matter whether manufacturer adopts CT Mode or ET Mode, the R&D level will increase with the increase of consumers’ green preferences. Since consumers prefer green, the R&D level of manufacturer adopting ET Mode is higher than that of manufacturer adopting CT Mode. The R&D level adopting CT Mode was not less than that of manufacturer adopting ET Mode. In addition, we also found that when consumers do not have green preference, the production quantity under the two technologies is equal.

**Fig 2:**
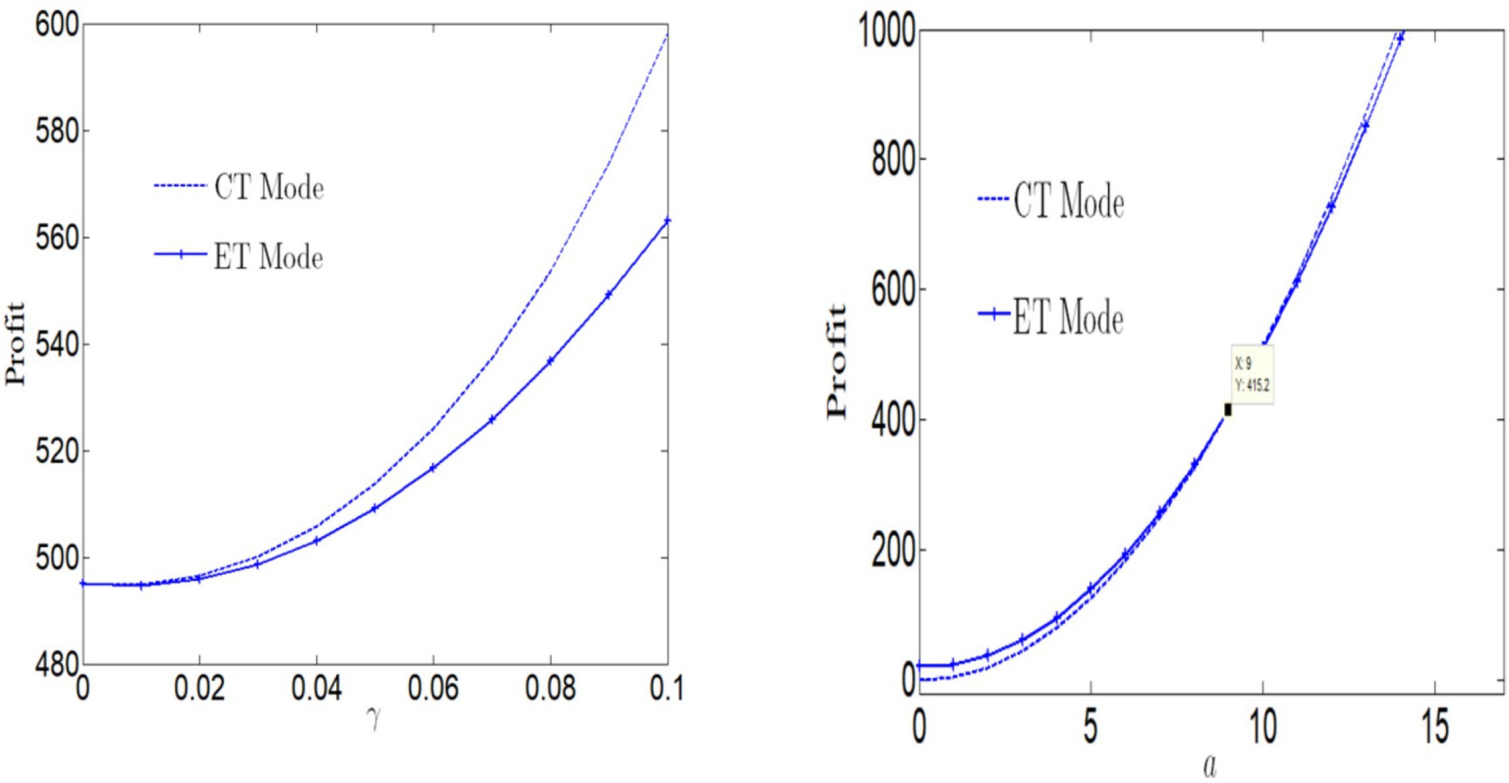
Impact of consumers’ green preference and market size on profits. (a) Impact of *γ* on profits. (b) Impact of *a* on profits.

Fig 2(A) analyzes the impact of consumer green preference on profits. No matter what technology manufacturer adopts, consumer green preference will increase profits. When consumers do not have green preference, we can find that the profit is equal when adopting two technologies. With the increase of consumers’ green preference, the manufacturer’s profit under CT Mode change more widely and is more vulnerable to the impact of consumers’ green preference. Therefore, the profit under CT Mode is greater than those under ET Mode, and with the increase of consumers’ green preferences, the profit gap between manufacturer under the two technology modes is growing.

From Fig 2(B), we can find that with the increase of market capacity, no matter what technology enterprises adopt, market capacity will increase manufacturer’s profit. When the market capacity is high, manufacturer’s profit under ET Mode is greater than that under CT Mode. When the market capacity is high, the adoption of the clean process is more conducive to the development of the enterprise. Therefore, when the market scale is large, compared with CT Mode, the CT Mode has become uneconomical.

**Fig 3:**
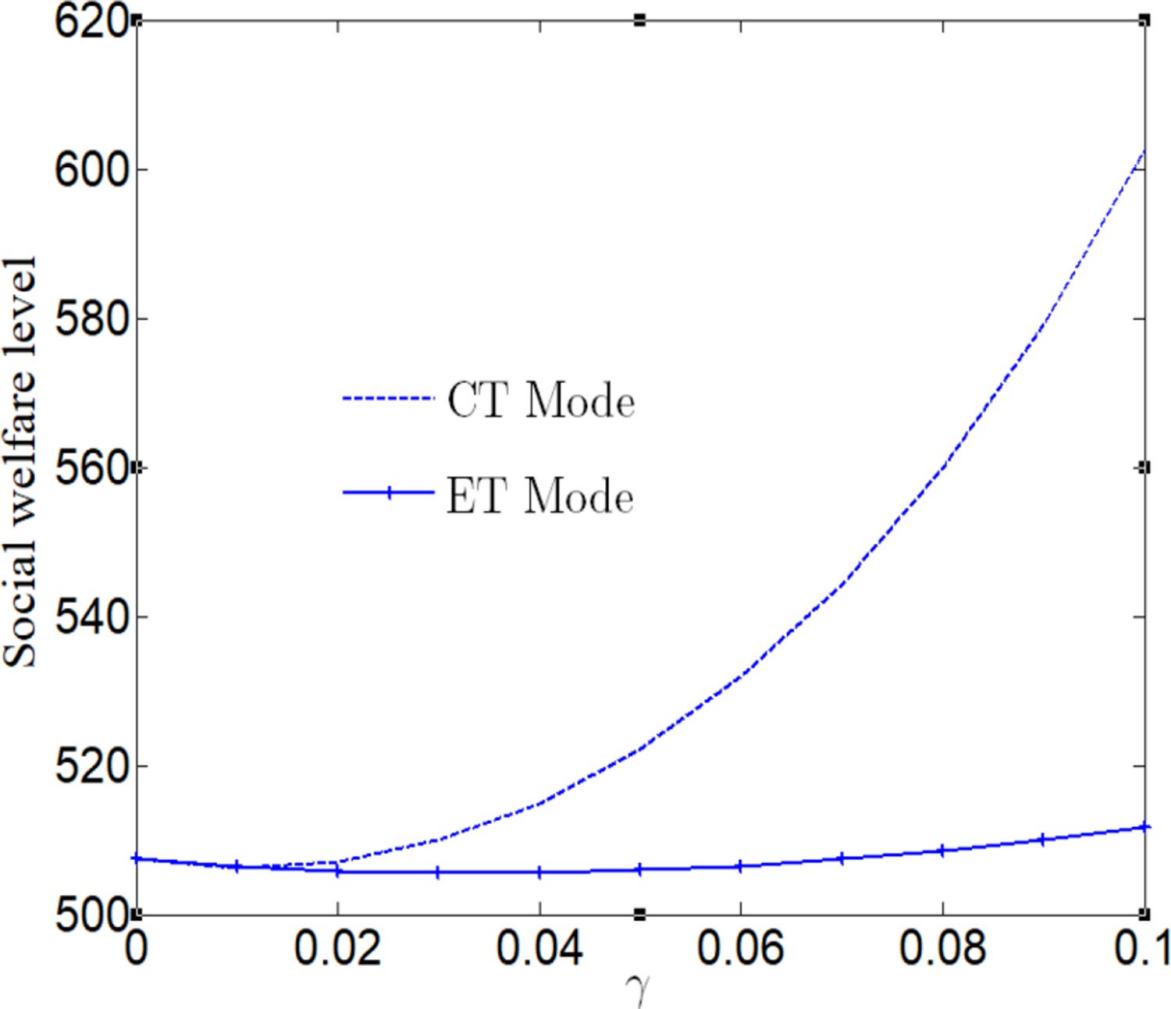
Impact of consumers’ green preference on social welfare.

It can be seen from Fig 3 that social welfare will decrease first and then increase with the increase of consumers’ green preference, regardless of whether CT Mode or ET Mode is used. However, if manufacturers use ET Mode, the range of social welfare changes is small, while manufacturers use CT Mode, the sensitivity of social welfare changes is greater. And with the increase of consumers’ green preference, the profit gap under the two modes is growing.

From Figs 1 to 3, we can draw the following conclusions.

First, whether using CT Mode or ET Mode, consumers’ green preference behavior can increase manufacturer’s profit, which is conducive to manufacturer’s development. However, when using CT Mode, manufacturer profit is more sensitive to consumers’ green preference behavior. Second, whether using CT Mode or ET Mode, when consumers’ green preference is small, consumers’ green preference is negatively related to social welfare. When consumers’ green preference is large, consumers’ green preference is conducive to increasing social welfare. However, when using CT Mode, social welfare is more vulnerable to the impact of consumers’ green preference behavior. Third, when the market volume is small, the manufacturer is more inclined to adopt ET Mode, and when the market volume is large, the manufacturer is more inclined to adopt CT Mode.

### 4.2 Corporate social responsibility without reward-penalty policy (NR Case)

When the manufacturer assumes social responsibility, its social responsibility level can be obtained according to relevant reports. For example, the Rating Standards for China’s Corporate Social Responsibility Report issued by the Chinese Academy of Social Sciences.

#### 4.2.1 CT Mode

When the manufacturer adopts CT Mode, the manufacturer’s decision objective function is

$$J_{c}^{NR}=\pi_{c}^{NR}+\pi^{E}=(a\theta -\theta q-\theta \gamma e)q-cq-\frac{1}{2}\eta_{c}k_{c}^{2}+t\frac{q^{2}}{2}$$ (24)

The overall revenue of the manufacturer consists of its economic revenue and the environmental revenue from its social responsibility, which indicates that the manufacturer takes the established social responsibility criteria into consideration in the decision-making process of operation. Now, our objective is to maximize $J_{c}^{NR}$ where ($q_{c}^{NR}$, $k_{c}^{NR}$) are decision variables. Differentiating $J_{c}^{NR}$ with respect to $q_{c}^{NR}$ and $k_{c}^{NR}$, we have

$$\frac{dJ_{c}^{NR}}{dq_{c}^{NR}}=(a\theta -\theta q-\theta \gamma e)-\theta q-c+tq,$$ (25)

$$\frac{dJ_{c}^{NR}}{dk_{c}^{NR}}=\theta \gamma l_{c}q-\eta_{c}k_{c}^{},$$ (26)

$$\frac{d^{2}J_{c}^{NR}}{dq_{c}^{NR}dk_{c}^{NR}}=\frac{d^{2}J_{c}^{NR}}{dk_{c}^{NR}dq_{c}^{NR}}=\theta \gamma l_{c},$$ (27)

$$\frac{d^{2}J_{c}^{NR}}{dq_{c}^{NR}{}^{2}}=-2\theta +t,$$ (28)

$$\frac{d^{2}J_{c}^{NR}}{dk_{c}^{NR}{}^{2}}=-\eta_{c}.$$ (29)

**Proposition 3:** When the manufacturer adopts CT Mode under NR case, the profit function of the manufacturer $J_{c}^{NR}$ attains maximum at ($q_{c}^{NR*}$, $k_{c}^{NR*}$) if *η*_*c*_(2*θ* ‒ *t*) > (*θγl*_*c*_)^2^ and *aθ* ‒ *θγm* > *c* hold. The optimal R&D level and yield under CT Mode are shown below.

$$k_{c}^{NR*}=\frac{\theta \gamma l_{c}(a\theta -c-\theta \gamma m)}{\eta_{c}(2\theta -t)-{\left(\theta \gamma l_{c}\right)}^{2}},$$ (30)

$$q_{c}^{NR*}=\frac{\eta_{c}(a\theta -\theta \gamma m-c)}{\eta_{c}(2\theta -t)-{\left(\theta \gamma l_{c}\right)}^{2}}.$$ (31)

**Proof:** For optimum values of $J_{c}^{NR}$, $\frac{dJ_{c}^{NR}}{dq_{c}^{NR}}=0$ and $\frac{dJ_{c}^{NR}}{dk_{c}^{NR}}=0$. Solving these equations, we have the required optimal values as follows:

$$k_{c}^{NR*}=\frac{\theta \gamma l_{c}(a\theta -c-\theta \gamma m)}{\eta_{c}(2\theta -t)-{\left(\theta \gamma l_{c}\right)}^{2}},$$

$$q_{c}^{NR*}=\frac{\eta_{c}(a\theta -\theta \gamma m-c)}{\eta_{c}(2\theta -t)-{\left(\theta \gamma l_{c}\right)}^{2}}.$$

Now the hessian matrix at ($q_{c}^{NR*}$, $k_{c}^{NR*}$) is

$$M=\left[\begin{array}{cc} \frac{d^{2}J_{c}^{NR}}{dq_{c}^{NR}{}^{2}} & \frac{d^{2}J_{c}^{NR}}{dk_{c}^{NR}dq_{c}^{NR}}\\ \frac{d^{2}J_{c}^{NR}}{dk_{c}^{NR}dq_{c}^{NR}} & \frac{d^{2}J_{c}^{NR}}{dk_{c}^{NR}{}^{2}} \end{array}\right]=\left[\begin{array}{cc} -2\theta +t & \theta \gamma l_{c}\\ \theta \gamma l_{c} & -\eta_{c} \end{array}\right].$$

Here, the principal minors are Δ_11_ = ‒2*θ*+*t*, Δ_22_ = ‒*η*_*c*_(‒2*θ*+*t*) ‒ (*θγl*_*c*_)^2^. If *η*_*c*_(2*θ*‒*t*) > (*θγl*_*c*_)^2^, the hessian matrix *M* at ($q_{c}^{NR*}$, $k_{c}^{NR*}$) is negative definite. Therefore, objective function $J_{c}^{NR}$ attains maximum at ($q_{c}^{NR*}$, $k_{c}^{NR*}$). Since the feasibility of the Eqs (30–31) require positive values of $q_{c}^{NR*}$ and $k_{c}^{NR*}$, *aθ* ‒ *θγm* > *c* must hold.

The optimal profit and social welfare level under CT Mode are

$$\pi_{c}^{NR}=[a\theta -c-\theta \gamma (m-l_{c}k_{c}^{NR}{}^{*})]q_{c}^{NR}{}^{*}-\theta q_{c}^{NR}{{}^{2}}^{*}-\frac{1}{2}\eta_{c}k_{c}^{NR2*},$$ (32)

$$F_{c}^{NR*}=\pi_{c}^{NR}+CS-veq_{c}^{NR*}=[a\theta -c-(\theta \gamma +v)(m-l_{c}k_{c}^{NR*})]q_{c}^{NR*}-\frac{1}{2}\eta_{c}k_{c}^{NR2*}+q_{c}^{NR2*}(\frac{1}{2}-\theta ).$$ (33)

#### 4.2.2 ET Mode

When the manufacturer adopts ET Mode, the manufacturer’s objective function is

$$J_{e}^{NR}=\pi_{e}^{NR}+\pi^{E}=[a\theta -\theta q-\theta \gamma (m-\frac{l_{e}k_{e}}{q})]q-cq-\frac{1}{2}\eta_{e}k_{e}^{2}+t\frac{q^{2}}{2}$$ (34)

Now, our objective is to maximize $J_{e}^{NR}$ where ($q_{e}^{NR}$, $k_{e}^{NR}$) are decision variables. Differentiating $J_{e}^{NR}$ with respect to $q_{e}^{NR}$ and $k_{e}^{NR}$, we have

$$\frac{dJ_{e}^{NR}}{dq_{e}^{NR}}=a\theta -2\theta q-\theta \gamma m-c+tq,$$ (35)

$$\frac{dJ_{e}^{NR}}{dk_{e}^{NR}}=\theta \gamma l_{e}-\eta_{e}k_{e}^{},$$ (36)

$$\frac{d^{2}J_{e}^{NR}}{dq_{e}^{NR}dk_{e}^{NR}}=\frac{d^{2}J_{e}^{NR}}{dk_{e}^{NR}dq_{e}^{NR}}=0,$$ (37)

$$\frac{d^{2}J_{e}^{NR}}{dq_{e}^{NR}{}^{2}}=-2\theta +t,$$ (38)

$$\frac{d^{2}J_{e}^{NR}}{dk_{e}^{NR}{}^{2}}=-\eta_{e}.$$ (39)

Now the hessian matrix at ($q_{e}^{NR*}$, $k_{e}^{NR*}$) is

$$M=\left[\begin{array}{cc} \frac{d^{2}J_{e}^{NR}}{dq_{e}^{NR}{}^{2}} & \frac{d^{2}J_{e}^{NR}}{dk_{e}^{NR}dq_{e}^{NR}}\\ \frac{d^{2}J_{e}^{NR}}{dk_{e}^{NR}dq_{e}^{NR}} & \frac{d^{2}J_{e}^{NR}}{dk_{e}^{NR}{}^{2}} \end{array}\right]=\left[\begin{array}{cc} -2\theta +t & 0\\ 0 & -\eta_{e} \end{array}\right]$$

**Proposition 4:** When the manufacturer adopts ET Mode under NR case, the profit function of the manufacturer $J_{e}^{NR}$ attains maximum at ($q_{e}^{NR*}$, $k_{e}^{NR*}$) if 2*θ*>*t* and *aθ*‒*θγm* > *c* hold. The optimal R&D level and yield under ET Mode are shown below.

$$q_{e}^{NR*}=\frac{a\theta -\theta \gamma m-c}{2\theta -t},$$ (40)

$$k_{e}^{NR*}=\frac{\theta \gamma l_{e}}{\eta_{e}}.$$ (41)

**Proof:** For optimum values of $J_{e}^{NR}$, $\frac{dJ_{e}^{NR}}{dq_{e}^{NR}}=0$ and $\frac{dJ_{e}^{NR}}{dk_{e}^{NR}}=0$. Solving these equations, we obtain the required optimal values as shown in Eqs (40) and (41). Now, the determinant of hessian matrix is $\frac{d^{2}J_{e}^{NR}}{dq_{e}^{NR}{}^{2}}\frac{d^{2}J_{e}^{NR}}{dk_{e}^{NR}{}^{2}}-{\left(\frac{d^{2}J_{e}^{NR}}{dq_{e}^{NR}dk_{e}^{NR}}\right)}^{2}=-\eta_{e}(-2\theta +t)$. If 2*θ*>*t*, objective function $J_{e}^{NR}$ is maximum, we have the maximum values of $J_{e}^{NR*}$. Since the feasibility of the Eq (40) requires positive value of $q_{e}^{NR*}$, *aθ*‒*θγm* > *c* must hold.

Similarly, the optimal profit and social welfare level under ET Mode are

$$\pi_{e}^{NR}=(a\theta -c+\beta e_{0}-\theta \gamma m)q+\theta \gamma l_{e}k_{e}-\frac{1}{2}\eta_{e}k_{e}^{2}-\theta q^{2},$$ (42)

$$F_{e}^{NR*}=\pi_{e}^{NR}-vq_{e}^{NR*}\frac{e^{2}}{2}=(a\theta -c-\theta \gamma m)q_{e}^{NR*}+\theta \gamma l_{e}k_{e}^{NR*}-\frac{1}{2}\eta_{e}k_{e}^{NR2*}+(\frac{1}{2}-\theta )q_{e}^{NR2*}-vq_{e}^{NR*}\frac{{\left(m-l_{e}k_{e}^{NR*}\right)}^{2}}{2}.$$ (43)

Based on the equilibrium results, the sensitivity of CRS is analyzed, and inference 3 and inference 4 can be obtained.

**Inference 3:**

If the manufacturer adopts CT Mode, the increase of corporate social responsibility improves the level of enterprise research and development $\frac{dK_{c}^{NR}}{dt}>0$, improves market demand $\frac{dq_{c}^{NR}}{dt}>0$, and increases consumer surplus $\frac{dCS_{e}^{NR}}{dt}>0$.

**Inference 4:** If the manufacturer adopts CT Mode, it will increase the market demand $\frac{dq_{e}^{NR}}{dt}>0$, reduce the product price $\frac{dp_{e}^{NR}}{dt}<0$, and increase the consumer surplus $\frac{dCS_{e}^{NR}}{dt}>0$. However, corporate social responsibility has nothing to do with the level of corporate research and development.

By comparing the equilibrium results under the two Cases that the manufacturer does not assume and assumes social responsibility, we get Theorem 3.

**Theorem 4: $k_{c}^{NR}>k_{c}^{NN}$**, $q_{c}^{NR}>q_{c}^{NN}$, $k_{e}^{NR}=k_{e}^{NN}$, $q_{e}^{NR}>q_{e}^{NN}$.

It is known from Theorem 3 that under CT Mode, the manufacturer’s social responsibility will improve the research and development level, reduce more pollutant emissions, thus increasing market demand and increasing consumer surplus. Under ET Mode, the manufacturer’s social responsibility will not affect the enterprise’s R&D level, but because of the increased cost of the enterprise due to the consideration of corporate social responsibility, the manufacturer can only achieve the optimal profit by increasing the yield.

The impacts of social responsibility parameter on optimal decisions and profits are investigated by using MATLAB in this section. Letting *a* = 10, *θ* = 20, *η*_*c*_ = 0.1, *η*_*e*_ = 0.13, *c* = 1, *v* = 0.1, *l*_*c*_ = 0.5, *l*_*e*_ = 2, *γ* = 0.05, *t* = 0.2. These parameter values satisfy the constraints of this paper.

**Fig 4:**
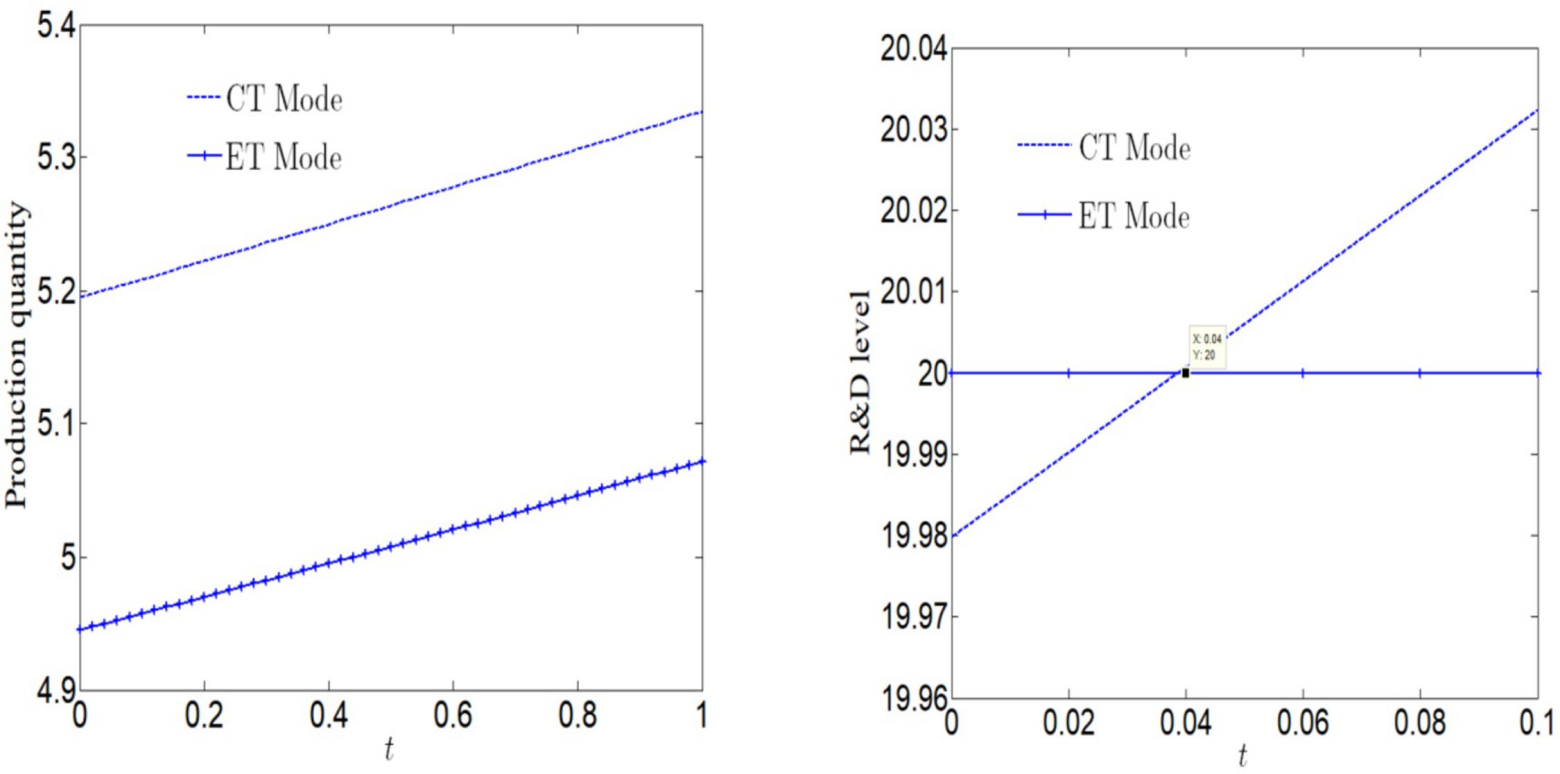
Impact of CSR on optimal decision-makings. (a) Impact of CSR on production quantity. (b) Impact of CSR on R&D level.

Fig 4(A) analyzes the impact of CSR on market demand. Regardless of whether the manufacturer adopt ET Mode or CT Mode, market demand increases with the increase of CSR. And we find that the market demand under CT Mode is greater than that under ET Mode. Fig 4(B) shows the impact of CSR on corporate R&D level. When corporate social responsibility *t* <0.04, the R&D level of enterprises adopting CT Mode is lower than that of the manufacturer adopting ET Mode. When *t* ≥0.04, the R&D level of the manufacturer adopting CT Mode is not less than that of the manufacturer adopting ET Mode.

**Fig 5:**
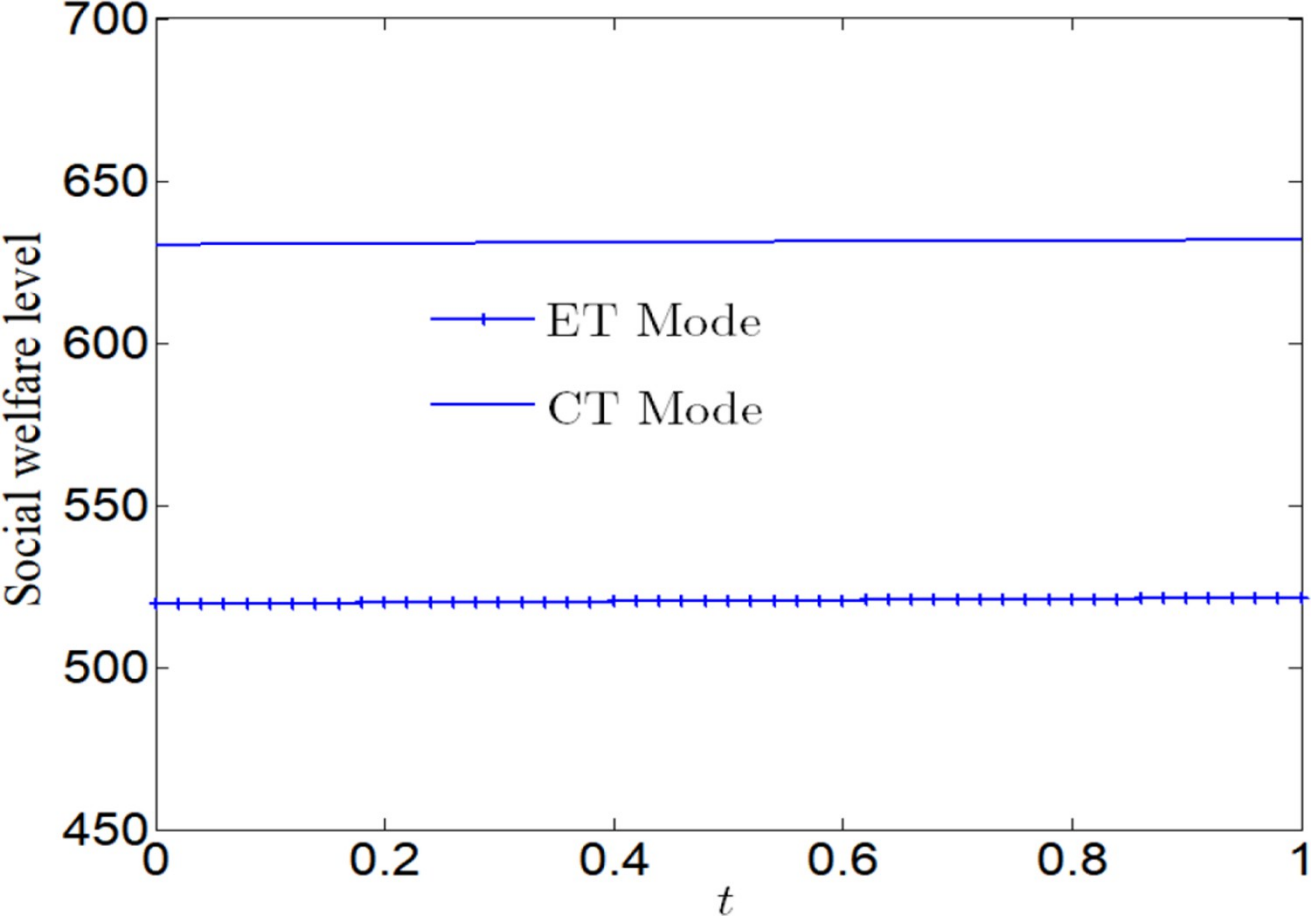
Impact of CSR on social welfare.

Fig 5 analyzes the impact of CSR on social welfare. Social welfare will increase with the increase of CSR, regardless of whether the manufacturer adopts ET Mode or CT Mode. And we find that the social welfare under CT Mode is greater than that under ET Mode.

**Fig 6:**
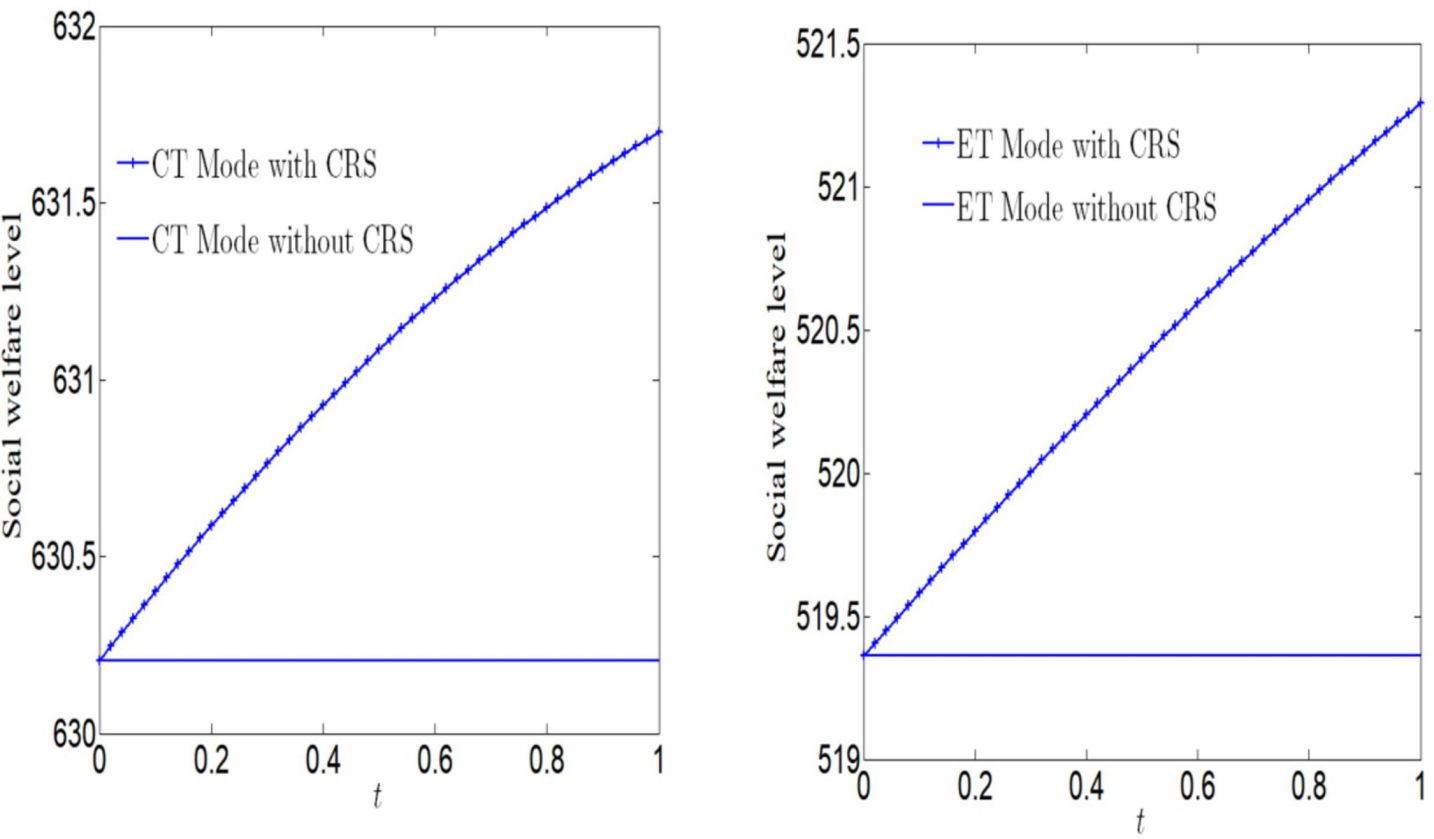
Impact of CSR on social welfare. (a) Impact of CSR on social welfare under CT Mode. (b) Impact of CSR on social welfare under ET Mode.

Fig 6(A) analyzes the impact of CSR on social welfare under CT Mode and ET Mode. No matter what emission reduction technology is used, social welfare will increase with the increase of CSR.

**Fig 7:**
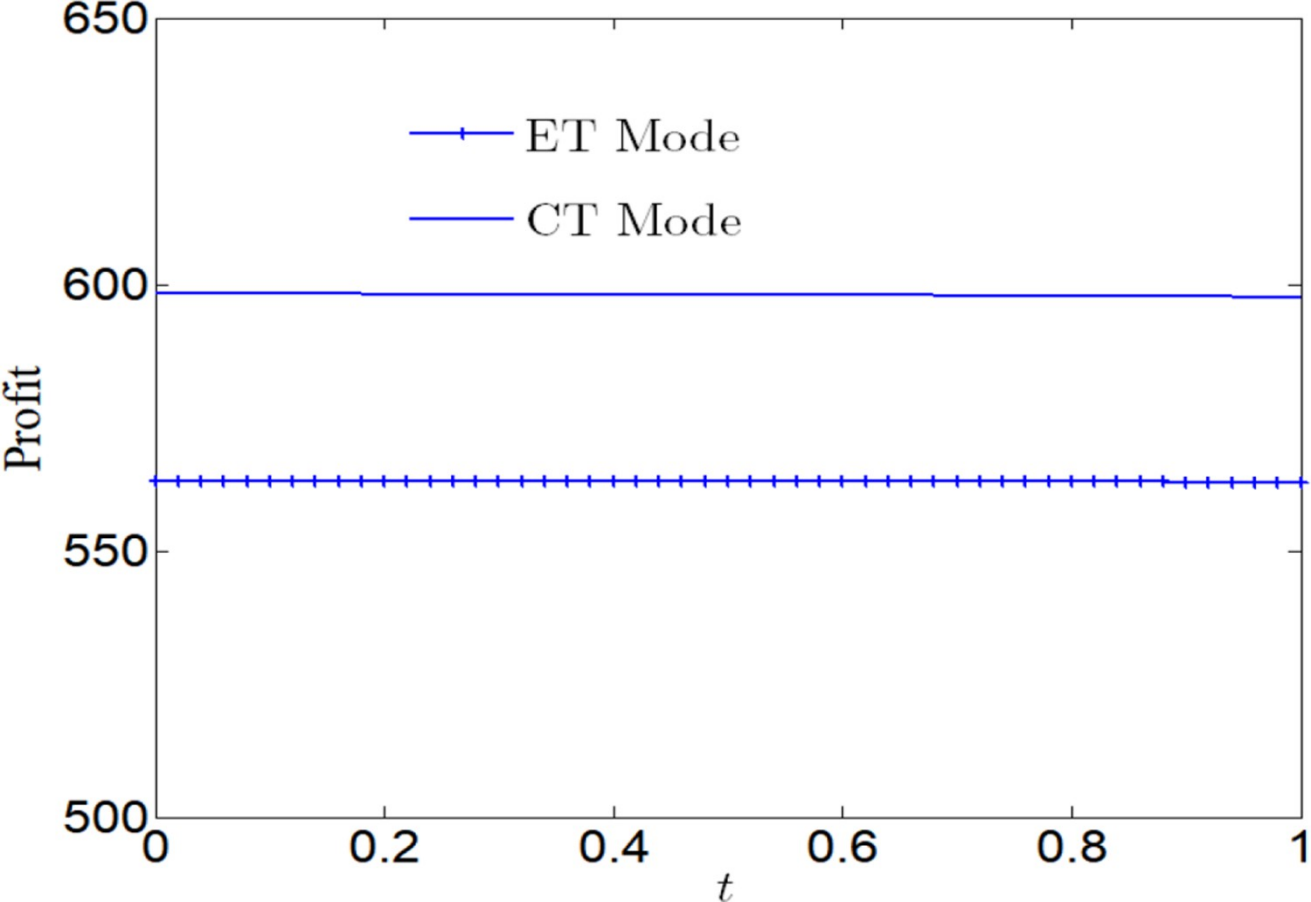
Impact of CSR on corporate profits.

Fig 7 analyzes the impact of CSR on profits. No matter whether the manufacturer adopts ET Mode or CT Mode, profits will decrease with the increase of CSR. And we find that the profit under CT Mode is greater than that under ET Mode.

**Fig 8:**
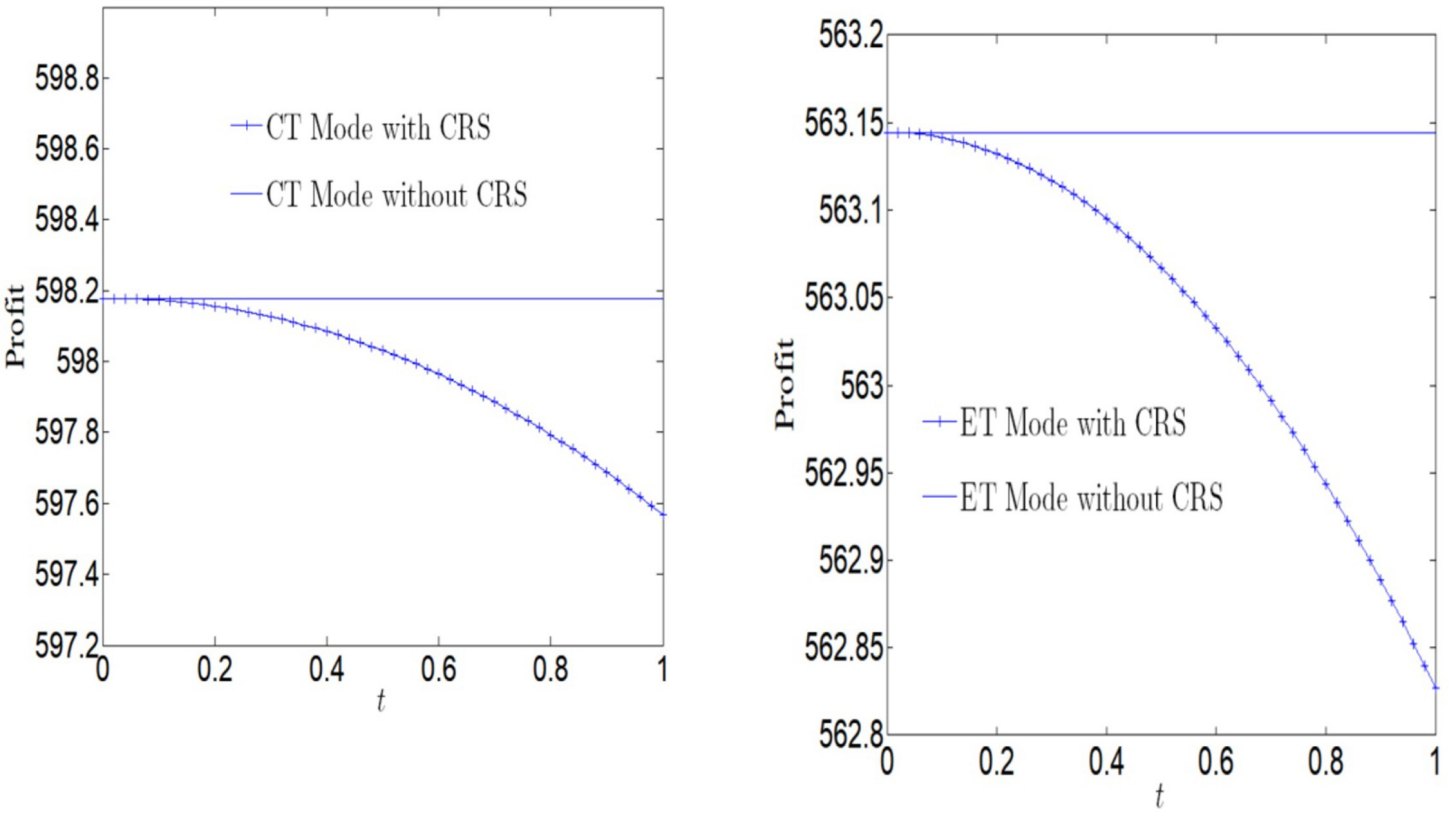
Impact of CSR on profits. (a) Impact of CSR on profits under CT Mode. (b) Impact of CSR on profits under ET Mode.

Fig 8 analyzes the impact of CSR on profits under CT Mode and ET Mode. No matter what emission reduction technology is used, profits will decrease with the increase of CSR. We find that the profit is more vulnerable to the impact of CSR under ET Mode.

No matter what kind of emission reduction technology is adopted, the higher the level of social responsibility, the more sensitive the environmental benefits are to consumer surplus, which is more conducive to encouraging the manufacturer to reduce pollutant emissions, expand market demand and increase consumer surplus from the perspective of consumer interests.

We find that the profit under CT Mode is always greater than that under ET Mode. No matter what emission reduction technology is adopted, the improvement of corporate social responsibility will lead to an increase in product prices. Consumers with green awareness preferences will tend to buy products with a higher degree of green, leading to an increase in market demand and social welfare.

However, the manufacturer’s social responsibility will damage the economic benefit of the manufacturer. As the manufacturer’s social responsibility is a conscious responsibility to consumers and the social environment, this behavior needs to pay a certain price, thus causing damage to its own economic benefit.

Based on the above, we draw the following conclusions:

First, no matter what emission reduction technology is used, corporate social responsibility will help to improve the level of social welfare, that is, greater than the social welfare without corporate social responsibility. Second, no matter whether a manufacturer adopts ET Mode or CT Mode, corporate social responsibility is not conducive to the increase of profit, that is, less than profit without corporate social responsibility. Under ET Mode, the profit is more likely to be affected by the level of corporate social responsibility. Third, when a manufacturer considers social responsibility, the adoption of clean technology is conducive to the profit and social welfare. Fourth, no matter whether a manufacturer adopt ET Mode or CT Mode, corporate social responsibility is conducive to improving market demand, and the market demand under CT Mode is greater than that under ET Mode. When a manufacturer adopts ET Mode, corporate social responsibility has nothing to do with the level of research and development. And with the increase of corporate social responsibility, the R&D level under CT Mode will exceed that under ET Mode.

Some scholars (i.e. Chen et al.) [28] pointed out that when enterprises have more funds, their survival pressure is less, which can promote enterprises to actively fulfill their social responsibilities. Since the manufacturer’s social responsibility will damage its economic benefit, the government can adopt corresponding subsidy mechanisms to ensure the manufacturer’s economic benefit. For example, the Chinese government will develop emission reduction mechanisms for high pollution and high energy consumption industries similar to the pharmaceutical manufacturing industry every year to encourage manufacturers to produce green products.

## 5. Reward-penalty policy (RP case)

We introduce not only a subsidy and reward mechanism, but also a punishment mechanism. On the one hand, to encourage manufacturers to reduce pollutant emissions, and encourage them to actively assume social responsibility and improve their level of social responsibility, the government can subsidize and reward manufacturers based on the emission reduction effect. On the other hand, in order to improve pollution control efficiency and environmental quality, a pollution control standard *e*_0_ is set. When the enterprise’s pollutant *e* is lower than the standard *e*_0_ set by the government, then the enterprise will receive a subsidy reward *βq*(*e*_0_‒*e*), and vice versa *βq*(*e*_0_‒*e*). Where, *β* > 0 is the government’s reward and punishment for the manufacturer’s emission reduction level. Next, we will explore the impact of this mechanism on enterprises’ adoption of clean process control technology and end-of-pipe pollution control technology.

### 5.1 CT Mode

When the manufacturer adopts CT Mode, the manufacturer’s decision objective function is

$$J_{c}^{PR}=\pi_{c}^{PR}+\pi^{E}=(a\theta -\theta q-\theta \gamma e)q-cq-\frac{1}{2}\eta_{c}k_{c}^{2}+\beta q(e_{0}-e)+t\frac{q^{2}}{2}$$ (44)

Now, our objective is to maximize $J_{c}^{PR}$ where ($q_{c}^{PR}$, $k_{c}^{PR}$) are decision variables. Differentiating $J_{c}^{PR}$ with respect to $q_{c}^{PR}$ and $k_{c}^{PR}$, we have

$$\frac{dJ_{c}^{PR}}{dq_{c}^{PR}}=a\theta -2\theta q-\theta \gamma e-c+tq+\beta (e_{0}-e),$$ (45)

$$\frac{dJ_{c}^{PR}}{dk_{c}^{PR}}=\theta \gamma l_{c}q-\eta_{c}k_{c}^{NN}+\beta ql_{c},$$ (46)

$$\frac{d^{2}J_{c}^{PR}}{dq_{c}^{PR}dk_{c}^{PR}}=\frac{d^{2}J_{c}^{PR}}{dk_{c}^{PR}dq_{c}^{PR}}=\theta \gamma l_{c}+\beta l_{c},$$ (47)

$$\frac{d^{2}J_{c}^{PR}}{dq_{c}^{PR}{}^{2}}=-2\theta +t,$$ (48)

$$\frac{d^{2}J_{c}^{PR}}{dk_{c}^{PR}{}^{2}}=-\eta_{c}.$$ (49)

Now the hessian matrix at ($q_{c}^{PR*}$, $k_{c}^{PR*}$) is

$$M=\left[\begin{array}{cc} \frac{d^{2}J_{c}^{PR}}{dq_{c}^{PR}{}^{2}} & \frac{d^{2}J_{c}^{PR}}{dk_{c}^{PR}dq_{c}^{PR}}\\ \frac{d^{2}J_{c}^{PR}}{dk_{c}^{PR}dq_{c}^{PR}} & \frac{d^{2}J_{c}^{PR}}{dk_{c}^{PR}{}^{2}} \end{array}\right]=\left[\begin{array}{cc} -2\theta +t & \theta \gamma l_{c}+\beta l_{c}\\ \theta \gamma l_{c}+\beta l_{c} & -\eta_{c} \end{array}\right].$$

**Proposition 5:** When the manufacturer adopts CT Mode under RP case, the profit function of the manufacturer $J_{c}^{PR}$ attains maximum at ($q_{c}^{PR*}$, $k_{c}^{PR*}$) if *t*<2*θ*,*η*_*c*_(2*θ* ‒ *t*) > (*θγ*+*β*)^2^*l*_*c*_^2^ and *aθ*‒*c*+*βe*_0_ > (*θγ*+*β*)*m* hold. The optimal R&D level and yield under CT Mode are shown below.

$$k_{c}^{PR*}=\frac{\left(\theta \gamma +\beta )l_{c}[a\theta -c+\beta e_{0}-(\theta \gamma +\beta )m\right]}{\eta_{c}(2\theta -t)-{\left(\theta \gamma +\beta \right)}^{2}l_{c}{}^{2}},$$ (50)

$$q_{c}^{PR*}=\frac{\eta_{c}[a\theta -c+\beta e_{0}-(\theta \gamma +\beta )m]}{\eta_{c}(2\theta -t)-{\left(\theta \gamma +\beta \right)}^{2}l_{c}{}^{2}},$$ (51)

**Proof:** Now for maximum value of $J_{c}^{PR}$, $\frac{dJ_{c}^{PR}}{dq_{c}^{PR}}=0=\frac{dJ_{c}^{PR}}{dk_{c}^{PR}}$. $\frac{dJ_{c}^{PR}}{dq_{c}^{PR}}=0$$\overset{yields}{\to }$$q_{c}^{PR*}$ $=\frac{\eta_{c}[a\theta -c+\beta e_{0}-(\theta \gamma +\beta )m]}{\eta_{c}(2\theta -t)-{\left(\theta \gamma +\beta \right)}^{2}l_{c}{}^{2}}$ and $\frac{dJ_{c}^{PR}}{dk_{c}^{PR}}=0$$\overset{yields}{\to }$$k_{c}^{PR*}$$=\frac{\left(\theta \gamma +\beta )l_{c}[a\theta -c+\beta e_{0}-(\theta \gamma +\beta )m\right]}{\eta_{c}(2\theta -t)-{\left(\theta \gamma +\beta \right)}^{2}l_{c}{}^{2}}$. Now at ($q_{c}^{PR*}$, $k_{c}^{PR*}$), $\frac{d^{2}J_{c}^{PR}}{dk_{c}^{PR}dq_{c}^{PR}}=\frac{d^{2}J_{c}^{PR}}{dq_{c}^{PR}dk_{c}^{PR}}=\theta \gamma l_{c}+\beta l_{c}$ and $\frac{d^{2}J_{c}^{PR}}{dq_{c}^{PR}{}^{2}}=-2\theta +t<0$ and $\frac{d^{2}J_{c}^{PR}}{dk_{c}^{PR}{}^{2}}=-\eta_{c}<0$. Here, the principal minors are Δ_11_ = ‒ 2*θ*+*t*, Δ_22_ = ‒*η*_*c*_(‒2θ+*t*)‒(*θγl*_*c*_+*βl*_*c*_)^2^. If *t* <2*θ* and *η*_*c*_(2*θ* ‒ *t*)>(*θγl*_*c*_)^2^, the hessian matrix M at ($q_{c}^{PR*}$, $k_{c}^{PR*}$) is negative definite. Therefore, objective function $J_{c}^{PR}$ attains maximum at ($q_{c}^{PR*}$, $k_{c}^{PR*}$). Since the feasibility of the Eqs (50–51) require positive values of $q_{c}^{PR*}$ and $k_{c}^{PR*}$, *aθ*‒*c*+*βe*_0_>(*θγ*+*β*)*m* must hold.

The optimal profit and social welfare level under CT Mode are

$$\pi_{c}^{PR}=[a\theta -c+\beta e_{0}-(\theta \gamma +\beta )(m-l_{c}k_{c}{}^{*})]q^{*}-\theta q^{2}{}^{*}-\frac{1}{2}\eta_{c}k_{c}^{2}{}^{*},$$ (52)

$$F_{c}^{PR*}=\pi_{c}^{PR}+CS-veq_{c}^{PR*}=[a\theta -c-(\theta \gamma +v)(m-l_{c}k_{c}^{PR*})]q_{c}^{PR*}-\frac{1}{2}\eta_{c}k_{c}^{PR2*}+q_{c}^{PR2*}(\frac{1}{2}-\theta ).$$ (53)

### 5.2 ET Mode

When the manufacturer adopts ET Mode, the manufacturer’s decision objective function is

$$J_{e}^{PR}=\pi_{e}^{PR}+\pi^{E}=[a\theta -\theta q-\theta \gamma (m-\frac{l_{e}k_{e}}{q})]q-cq-\frac{1}{2}\eta_{e}k_{e}^{2}+\beta q[e_{0}-(m-\frac{l_{e}k_{e}}{q})]+t\frac{q^{2}}{2}$$ (54)

Taking the derivative of $J_{e}^{PR}$ with respect to $q_{e}^{PR}$ and $k_{e}^{PR}$, we have

$$\frac{dJ_{e}^{PR}}{dq_{e}^{PR}}=a\theta -2\theta q-\theta \gamma m-c+tq+\beta (e_{0}-m),$$ (55)

$$\frac{dJ_{e}^{PR}}{dk_{e}^{PR}}=\theta \gamma l_{e}-\eta_{e}k_{e}^{}+\beta l_{e},$$ (56)

$$\frac{d^{2}J_{e}^{PR}}{dq_{e}^{PR}dk_{e}^{PR}}=\frac{d^{2}J_{e}^{PR}}{dk_{e}^{PR}dq_{e}^{PR}}=0,$$ (57)

$$\frac{d^{2}J_{e}^{PR}}{dq_{e}^{PR2}}=-2\theta +t,$$ (58)

$$\frac{d^{2}J_{e}^{PR}}{dk_{e}^{PR2}}=-\eta_{e}.$$ (59)

Now, the hessian matrix at ($q_{e}^{PR*}$, $k_{e}^{PR*}$) is

$$M=\left[\begin{array}{cc} \frac{d^{2}J_{e}^{PR}}{dq_{e}^{PR}{}^{2}} & \frac{d^{2}J_{e}^{PR}}{dk_{e}^{PR}dq_{e}^{PR}}\\ \frac{d^{2}J_{e}^{PR}}{dq_{e}^{PR}dk_{e}^{PR}} & \frac{d^{2}J_{e}^{PR}}{dk_{e}^{PR}{}^{2}} \end{array}\right]=\left[\begin{array}{cc} -2\theta +t & 0\\ 0 & -\eta_{e} \end{array}\right]$$

**Proposition 6:** When the manufacturer adopts ET Mode under RP case, the profit function of the manufacturer $J_{e}^{PR}$ attains maximum at ($q_{e}^{PR*}$, $k_{e}^{PR*}$) if *aθ*‒*θγm*‒*c*+*β*(*e*_0_‒*m*)>0 and 2*θ*>*t* hold. The optimal R&D level and yield under ET Mode are shown below.

$$q_{e}^{PR*}=\frac{a\theta -\theta \gamma m-c+\beta (e_{0}-m)}{2\theta -t},$$ (60)

$$k_{e}^{PR*}=\frac{(\theta \gamma +\beta )l_{e}}{\eta_{e}}.$$ (61)

**Proof:** Now for maximum value of $J_{e}^{PR}$, $\frac{dJ_{e}^{PR}}{dq_{e}^{PR}}=0=\frac{dJ_{e}^{PR}}{dk_{e}^{PR}}$. $\frac{dJ_{e}^{PR}}{dq_{e}^{PR}}=0$$\overset{yields}{\to }$$q_{e}^{PR*}$ $=\frac{a\theta -\theta \gamma m-c+\beta (e_{0}-m)}{2\theta -t}$ and $\frac{dJ_{e}^{PR}}{dk_{e}^{PR}}=0$$\overset{yields}{\to }$$k_{e}^{PR*}$$=\frac{(\theta \gamma +\beta )l_{e}}{\eta_{e}}$. Now at ($q_{e}^{PR*}$, $k_{e}^{PR*}$), $\frac{d^{2}J_{e}^{PR}}{dk_{e}^{PR}dq_{e}^{PR}}=\frac{d^{2}J_{e}^{PR}}{dq_{e}^{PR}dk_{e}^{PR}}=0$. The determinant of hessian matrix is $\frac{d^{2}J_{e}^{PR}}{dq_{e}^{PR}{}^{2}}\frac{d^{2}J_{e}^{PR}}{dk_{e}^{PR}{}^{2}}-{\left(\frac{d^{2}J_{e}^{PR}}{dq_{e}^{PR}dk_{e}^{PR}}\right)}^{2}=-\eta_{e}(-2\theta +t)$. If *η*_*e*_(2*θ*‒*t*)>0, objective function $J_{e}^{PR}$ is maximum, and we have the maximum value of $J_{e}^{PR}$. Since the feasibility of the Eq (60) requires positive value of $q_{e}^{PR*}$, *aθ*‒*θγm*‒*c*+*β*(*e*_0_‒*m*)>0 must hold.

The optimal profit and social welfare level under ET Mode are

$$\pi_{e}^{PR}=[a\theta -c+\beta e_{0}-(\theta \gamma +\beta )m]q+(\theta \gamma +\beta )l_{e}k_{e}-\frac{1}{2}\eta_{e}k_{e}^{2}-\theta q^{2},$$ (62)

$$F_{e}^{PR*}=\pi_{e}^{PR}-vq_{e}^{PR*}\frac{e^{2}}{2}=(a\theta -c-\theta \gamma m)q_{e}^{PR*}+\theta \gamma l_{e}k_{e}^{PR*}-\frac{1}{2}\eta_{e}k_{e}^{PR2*}+(\frac{1}{2}-\theta )q_{e}^{PR2*}-vq_{e}^{PR*}\frac{{\left(m-l_{e}k_{e}^{PR*}\right)}^{2}}{2}.$$ (63)

**Theorem 2:** Under RP case, only when *t* <2*θ*, the optimal R&D level and yield under CT Mode are:

$$k_{c}^{PR}=\frac{\left(\theta \gamma +\beta )l_{c}[(a\theta -c+\beta e_{0})-(\theta \gamma +\beta )m\right]}{\eta_{c}(2\theta -t)-{\left(\theta \gamma +\beta \right)}^{2}l_{c}{}^{2}},$$

$$q_{c}^{PR}=\frac{\eta_{c}[a\theta -(\theta \gamma +\beta )m-c+\beta e_{0}]}{\eta_{c}(2\theta -t)-{\left(\theta \gamma +\beta \right)}^{2}l_{c}{}^{2}}.$$

The optimal R&D level and yield under ET Mode are:

$$k_{e}^{PR}=\frac{(\theta \gamma +\beta )l_{e}}{\eta_{e}},$$

$$q_{e}^{PR}=\frac{a\theta -\theta \gamma m-c+\beta (e_{0}-m)}{2\theta -t}.$$

Based on the equilibrium results, the sensitivity of CRS is analyzed, and inference 5 and inference 6 can be obtained.

**Inference 5:** If the manufacturer adopts clean process technology, when *m*_2_*l*_*c*_^2^*β*^2^ ‑ 2*m*_1_*l*_*c*_^2^*β* + *M*_3_>0, the reward-penalty policy will help to increase market demand $\frac{dq_{c}^{PR}}{d\beta }>0$ and increase consumer surplus $\frac{dCS_{c}^{PR}}{d\beta }<0$, and vice versa. When 2*bM*_4_ > *b*^2^*M*_5_ + *M*_6_, the reward-penalty policy helps to improve the level of green R&D $\frac{dk_{c}^{PR}}{d\beta }>0$, and vice versa $\frac{dk_{c}^{PR}}{d\beta }<0$.

Where, *m*_1_ = *aθ*‒*θγm* ‒ *c*, *m*_2_ = *m*‒*e*_0_, *M*_3_ = 2*m*_2_*η*_2_*θ*‒*m*_2_*η*_2_*t* ‒ *m*_2_(*θγl*_*c*_)^2^‒2*l*_c_^2^*m*_1_ *θγ*, *M*_4_ = 2*m*_2_*η*_2_*θ*‒*m*_1_*θγl*_c_^2^ ‒ *m*_2_*η*_2_*t* ‒ *m*_2_(*θγl*_*c*_)^2^,*M*_5_ = *m*_2_*θγl*_c_^2^+*m*_1_*l*_c_^2^, *M*_6_ = 2*m*_1_*η*_*c*_*θ*‒*m*_1_*η*_*c*_*t* + *m*_1_(*θγl*_*c*_)^2^‒2*m*_2_ *θ*^2^γ*η*_*c*_ + *m*_2_ *θγη*_*c*_*t* + *m*_2_(*θγ*)^3^*l*_*c*_^2^.

**Inference 6:** If the manufacturer adopts ET Mode, it will improve the level of green R&D $\frac{dk_{e}^{PR}}{d\beta }>0$; When *e*_0_ < *m*, it reduces the market demand $\frac{dq_{e}^{PR}}{d\beta }<0$ and the consumer surplus $\frac{dCS_{e}^{PR}}{d\beta }<0$.

Next, we will compare the optimal decision, profit and social welfare of the two pollution control models under different circumstances. As shown in Table 1.

**Table 1:** Comparison of optimal decision, profit and social welfare under different cases.

Mode	yield	R&D	Profit	Social welfare
NN case (No CSR without reward-penalty policy)				
CT	$q_{c}^{NN*}=\frac{\eta_{c}[a\theta -\theta \gamma m-c]}{\eta_{c}(2\theta )-{\left(\theta \gamma \right)}^{2}l_{c}{}^{2}}$	$k_{c}^{NN*}=\frac{\theta \gamma l_{c}[(a\theta -c)-\theta \gamma m]}{\eta_{c}(2\theta )-{\left(\theta \gamma \right)}^{2}l_{c}{}^{2}}$	$\begin{array}{l} \pi_{c}^{NN*}=[a\theta -c-\theta \gamma (m-l_{c}k_{c}^{NN}{}^{*})]q_{c}^{NN}{}^{*}\\ -\theta q_{c}^{NN}{{}^{*}}^{2}-\frac{1}{2}\eta_{c}k_{c}^{NN*2} \end{array}$	$\begin{array}{l} F_{c}^{NN*}=[a\theta -c-(\theta \gamma +v)(m-l_{c}k_{c}^{NN*})]q_{c}^{NN*}\\ -\frac{1}{2}\eta_{c}k_{c}^{NN*2}+q_{c}^{NN*2}(\frac{1}{2}-\theta ) \end{array}$
ET	$q_{e}^{NN*}=\frac{a\theta -\theta \gamma m-c}{2\theta }$	$k_{e}^{NN*}=\frac{\theta \gamma l_{e}}{\eta_{e}}$	$\begin{array}{l} \pi_{c}^{NN*}=[a\theta -c-\theta \gamma (m-l_{c}k_{c}^{NN}{}^{*})]q_{c}^{NN}{}^{*}\\ -\theta q_{c}^{NN}{{}^{*}}^{2}-\frac{1}{2}\eta_{c}k_{c}^{NN*2} \end{array}$	$\begin{array}{l} F_{e}^{NN*}=(a\theta -c-\theta \gamma m)q_{e}^{NN*}+\theta \gamma l_{e}k_{e}^{NN*}-\frac{1}{2}\eta_{e}k_{e}^{NN*2}\\ +(\frac{1}{2}-\theta )q_{e}^{NN*2}-vq_{e}^{NN*}\frac{{\left(m-l_{e}k_{e}^{NN*}\right)}^{2}}{2} \end{array}$
NR case (CSR without reward-penalty policy)				
CT	$q_{c}^{NR*}=\frac{\eta_{c}(a\theta -\theta \gamma m-c)}{\eta_{c}(2\theta -t)-{\left(\theta \gamma l_{c}\right)}^{2}}$	$k_{c}^{NR*}=\frac{\theta \gamma l_{c}(a\theta -c-\theta \gamma m)}{\eta_{c}(2\theta -t)-{\left(\theta \gamma l_{c}\right)}^{2}}$	$\begin{array}{l} \pi_{c}^{NR*}=[a\theta -c-\theta \gamma (m-l_{c}k_{c}^{NR}{}^{*})]q_{c}^{NR}{}^{*}\\ -\theta q_{c}^{NR}{}^{*2}-\frac{1}{2}\eta_{c}k_{c}^{NR*2} \end{array}$	$\begin{array}{l} F_{c}^{NR*}=[a\theta -c-(\theta \gamma +v)(m-l_{c}k_{c}^{NR*})]q_{c}^{NR*}\\ -\frac{1}{2}\eta_{c}k_{c}^{NR*2}+q_{c}^{NR*2}(\frac{1}{2}-\theta ) \end{array}$
ET	$q_{e}^{NR*}=\frac{a\theta -\theta \gamma m-c}{2\theta -t}$	$k_{e}^{NR*}=\frac{\theta \gamma l_{e}}{\eta_{e}}$	$\begin{array}{l} \prod_{e}^{NR*}=(a\theta -c-\theta \gamma m)q_{e}{}^{NR*}\\ +\theta \gamma l_{e}k_{e}-\frac{1}{2}\eta_{e}k_{e}^{NR*2}+(\frac{t}{2}-\theta )q_{e}{{}^{NR*}}^{2} \end{array}$	$\begin{array}{l} F_{e}^{NR*}=(a\theta -c-\theta \gamma m)q_{e}^{NR*}+\theta \gamma l_{e}k_{e}^{NR*}\\ -\frac{1}{2}\eta_{e}k_{e}^{NR*2}+(\frac{1}{2}-\theta )q_{e}^{NR*2}-vq_{e}^{NR*}\frac{{\left(m-l_{e}k_{e}^{NR*}\right)}^{2}}{2} \end{array}$
RP case (CSR with reward-penalty policy)				
CT	$q_{c}^{PR*}=\frac{\eta_{c}\left[\begin{array}{l} a\theta -c+\beta e_{0}\\ -(\theta \gamma +\beta )m \end{array}\right]}{\eta_{c}(2\theta -t)-{\left(\theta \gamma +\beta \right)}^{2}l_{c}{}^{2}}$	$k_{c}^{PR*}=\frac{(\theta \gamma +\beta )l_{c}\left[\begin{array}{l} \left(a\theta -c+\beta e_{0}\right)\\ -(\theta \gamma +\beta )m \end{array}\right]}{\eta_{c}(2\theta -t)-{\left(\theta \gamma +\beta \right)}^{2}l_{c}{}^{2}}$	$\begin{array}{l} \pi_{c}^{PR*}=[a\theta -c+\beta e_{0}-(\theta \gamma +\beta )(m-l_{c}k_{c}{}^{*})]q^{*}\\ -\theta q^{*}{}^{2}-\frac{1}{2}\eta_{c}k_{c}^{*2} \end{array}$	$\begin{array}{l} F_{c}^{PR*}=[a\theta -c-(\theta \gamma +v)(m-l_{c}k_{c}^{PR*})]q_{c}^{PR*}\\ -\frac{1}{2}\eta_{c}k_{c}^{PR*2}+q_{c}^{PR*2}(\frac{1}{2}-\theta ) \end{array}$
ET	$q_{e}^{PR*}=\frac{a\theta -\theta \gamma m-c+\beta (e_{0}-m)}{2\theta -t}$	$k_{e}^{PR*}=\frac{(\theta \gamma +\beta )l_{e}}{\eta_{e}}$	$\begin{array}{l} \pi_{e}^{PR*}=[a\theta -c+\beta e_{0}-(\theta \gamma +\beta )m]q_{e}^{PR*}\\ +(\theta \gamma +\beta )l_{e}k_{e}^{PR*}-\frac{1}{2}\eta_{e}k_{e}^{PR*2}-\theta q_{e}^{PR*}{}^{2} \end{array}$	$\begin{array}{l} F_{e}^{PR*}=(a\theta -c-\theta \gamma m)q_{e}^{PR*}+\theta \gamma l_{e}k_{e}^{PR*}\\ -\frac{1}{2}\eta_{e}k_{e}^{PR*2}+(\frac{1}{2}-\theta )q_{e}^{PR*2}-vq_{e}^{PR*}\frac{{\left(m-l_{e}k_{e}^{PR*}\right)}^{2}}{2} \end{array}$

The impacts of related parameters on optimal decisions and profits are investigated by using MATLAB in this section. Letting *a* = 10, *θ* = 20, *η*_*c*_ = 0.1, *η*_*e*_ = 0.13, *c* = 1, *v* = 0.1, *l*_*c*_ = 0.5, *l*_*e*_ = 2, *γ* = 0.1, *t* = 1.

**Fig 9:**
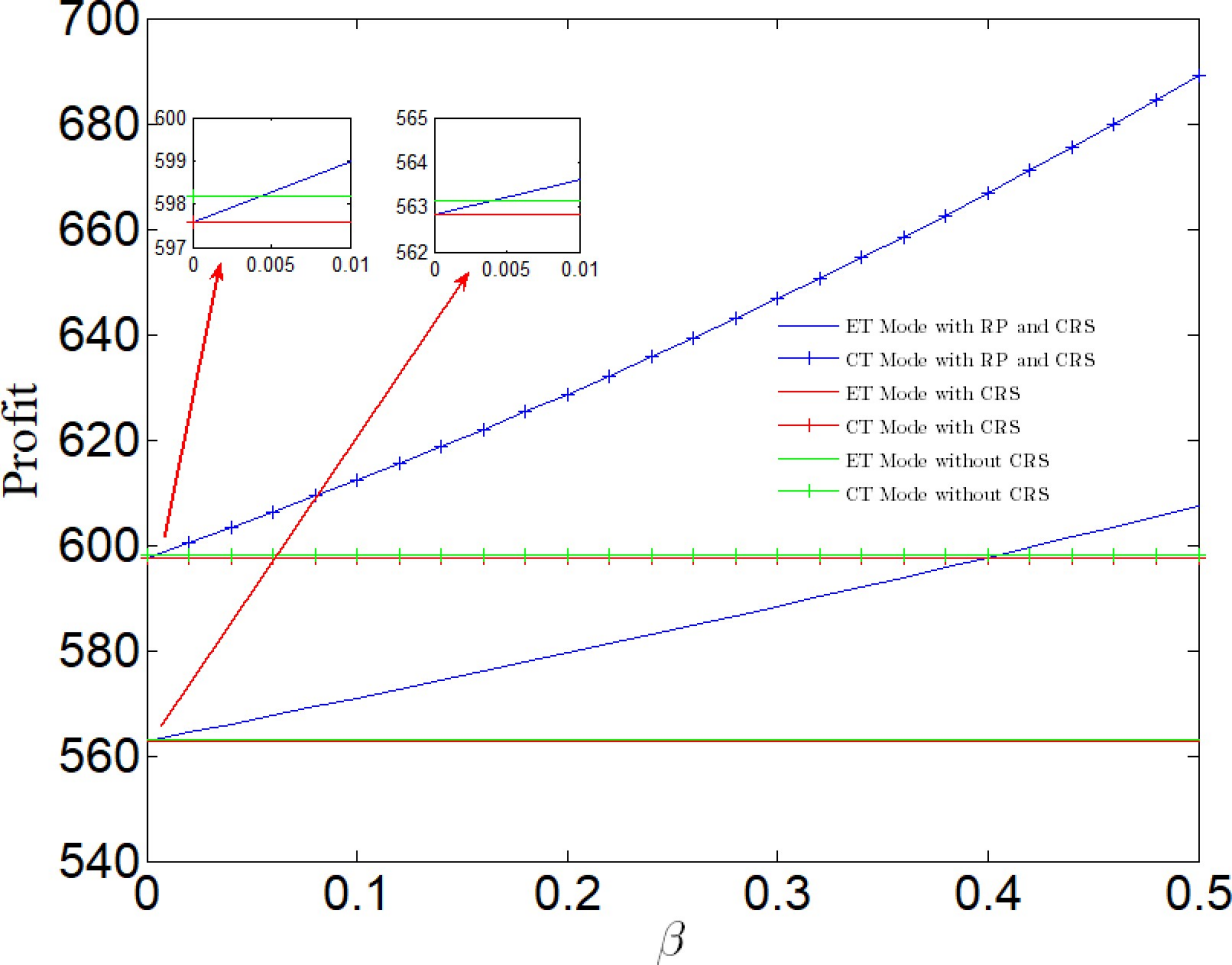
Impact of rewards and punishments on the manufacturer.

Fig 9 analyzes the impact of rewards and punishments on profits. We can find that no matter which pollution control technology is adopted, the profits increase with the increase of rewards and punishments. When the reward and punishment are small, the profit may be less than that under NN case, but it is always greater than that under NR case. Because the reward-penalty policy is so weak that it can not stimulate manufacturers to consider corporate social responsibility. From section 5, we know that when manufacturers consider CSR, social welfare will increase, but profits will decrease. Therefore, from the perspective of pure economic profit, manufacturers have no incentive to consider corporate social responsibility. In this context, we can only turn to the government for help. The goal of the government is to improve social welfare. Therefore, only when the government provides certain subsidies to make the profits of enterprises larger than those under NN case, the reward-penalty policy can stimulate enterprises to assume CSR and play an incentive role. Therefore, from Fig 9, we find that the greater the reward and punishment, the gap between the profit under NN case and that under RP case is growing.

**Fig 10:**
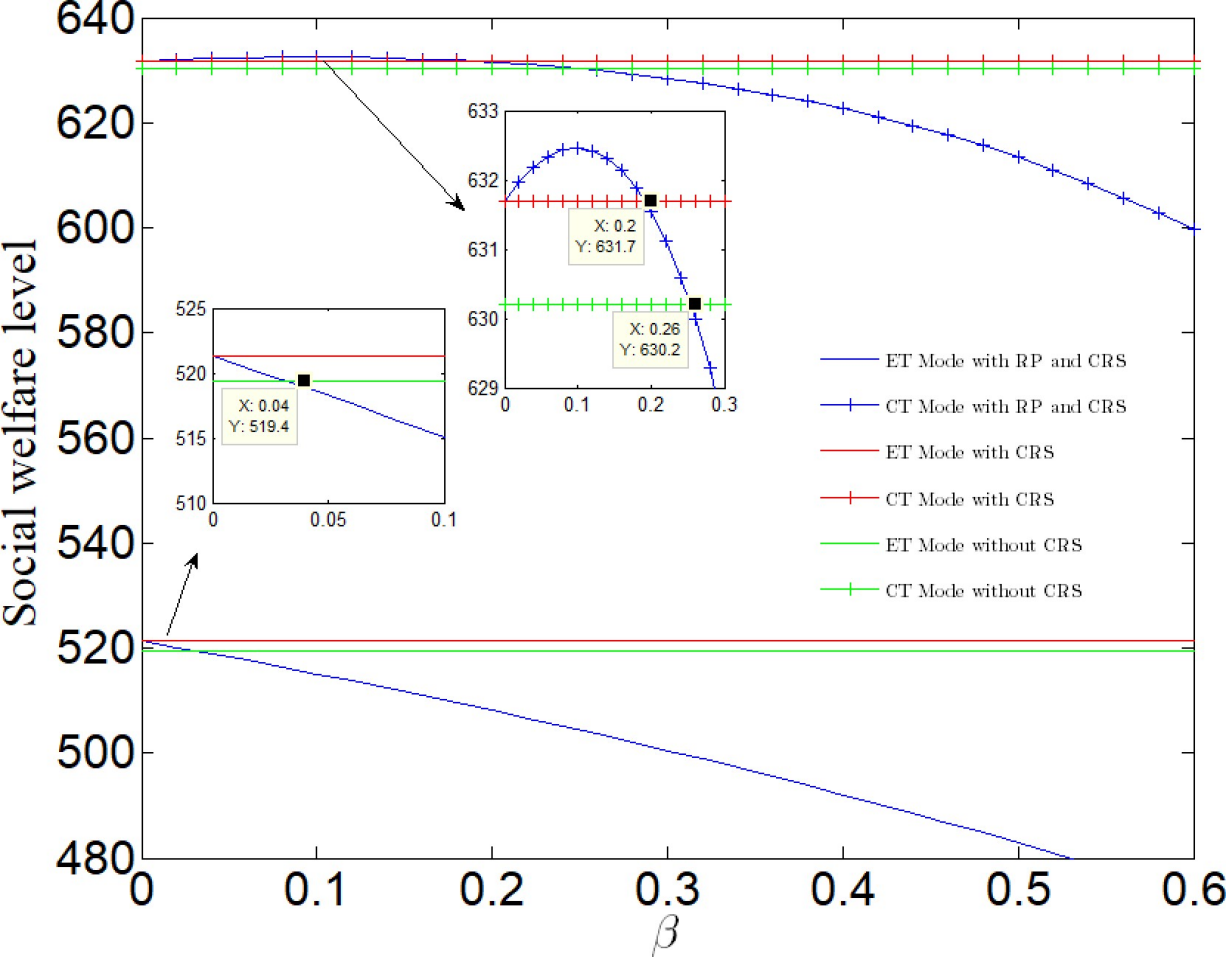
Impact of rewards and punishments on social welfare.

It can be seen from Fig 10 that if a manufacturer adopts CT Mode, the social welfare will first increase and then decrease with the increase of reward and punishment. When 0 < *β* < 0.2, the social welfare will be greater than that without reward-penalty policy. When 0.2 < *β* < 0.26, the social welfare will be greater than that under NN case, and less than that under NR case. When *β* > 0.26, the social welfare will be the lowest in several cases; If the manufacturer adopts ET Mode, the social welfare will decrease with the increase of rewards and punishments. When 0 < *β* < 0.04, the social welfare is greater than that under NN case, and less than that under NR case. When *β* > 0.04, the social welfare is the lowest in several cases.

The purpose of the government to establish a reward-penalty policy is to achieve a win-win situation for social welfare and enterprise profits, which can produce incentive effects for enterprises. It can be seen from Section 4–5 and Fig 9 that if an enterprise considers social responsibility, social welfare will be improved. Under RP case, only when the enterprise adopts CT Mode and 0.05< *β* < 0.26, the reward-penalty policy can achieve a win-win situation for social welfare and enterprise profits, that is, both are greater than the value under NN case and NR case; Among them, when 0.05< *β* < 0.2, the social welfare is optimal; When 0.2< *β* < 0.26, the profit is optimal. When *β* > 0.26, the reward-penalty policy can not achieve a win-win situation of social welfare and enterprise profits, but can only effectively stimulate enterprises.

**Fig 11:**
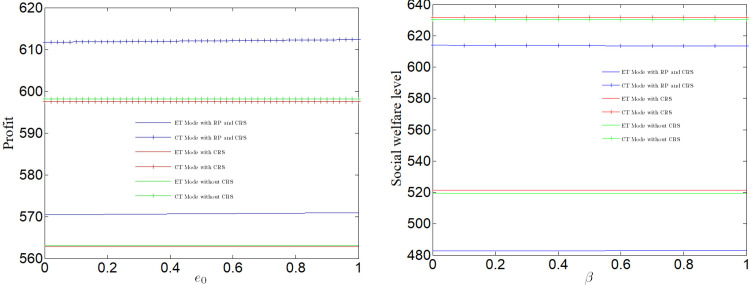
Impact of standard *e*_0_ on profit and social welfare.

Fig 11(A) analyzes the impact of the standard value *e*_0_ on profit. It can be found that the manufacturer profit increase with the increase of *e*_0_ regardless of whether CT Mode or ET Mode. The bigger *e*_0_ is, the looser the government’s pollution control is, the lower the enterprise’s R&D cost is, and the higher the enterprise’s profit is. The lower *e*_0_ is, the stricter the government’s pollution control is. Fig 11(B) analyzes the impact of *e*_0_ on social welfare. It can be found that whether CT Mode or ET Mode, the increase of *e*_0_ has little impact on social welfare.

**Fig 12:**
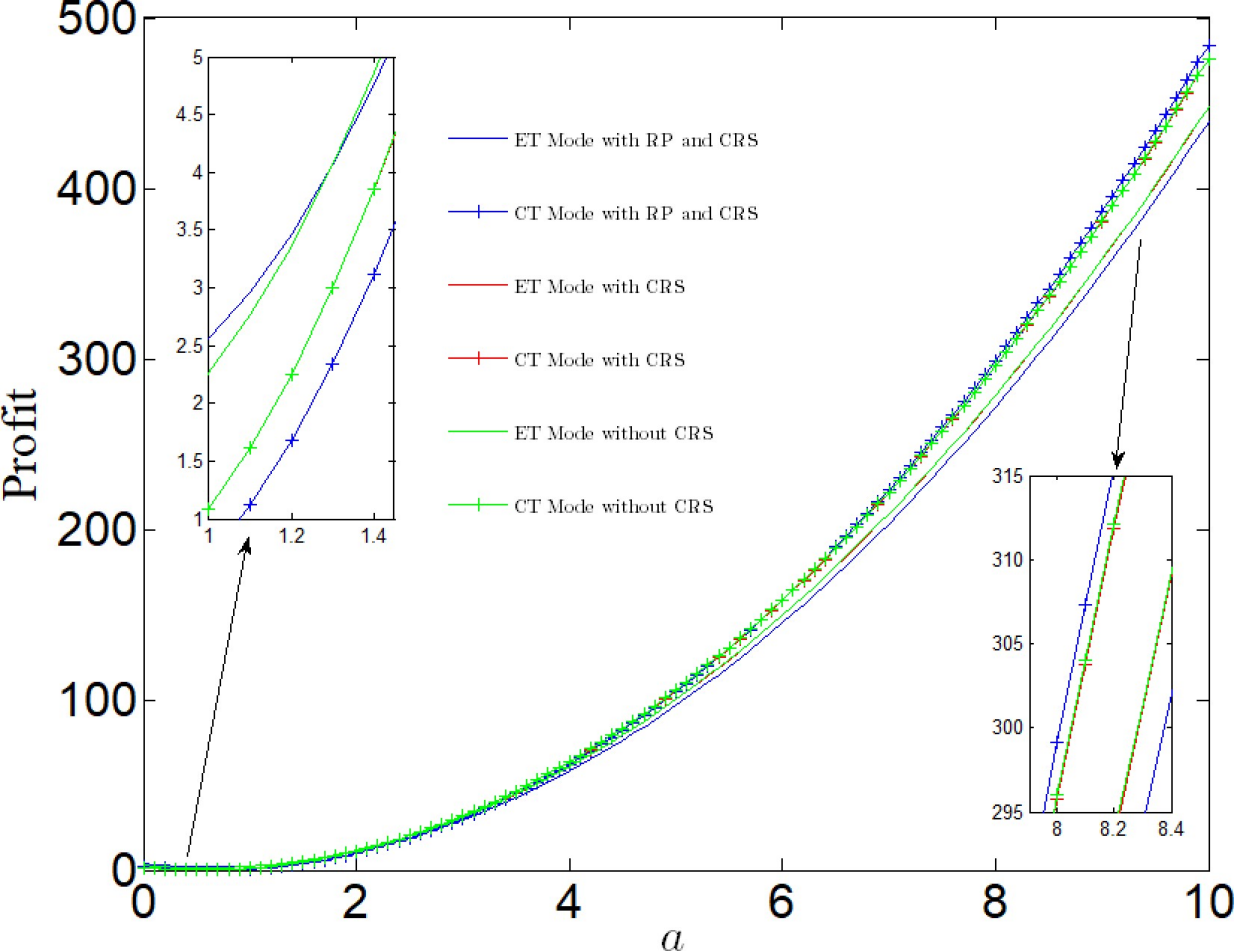
Impact of market size on profit.

Fig 12 analyzes the impact of market size on profit. No matter whether CT Mode or ET Mode, profits will decrease first and then increase with the increase of market size. That is, when the market size is small, profit decreases with the increase of the market size. When the market size is large, profit increases with the increase of the market size. When the market size is small, the increase of the market size will lead to a smaller increase in revenue than the increase in R&D costs, leading to a decline in profit. When the market size is large, the increase in market size leads to an increase in revenue greater than the increase in R&D costs, which increases profits.

We also find that when the market size is small, profits under CT Mode are less than those ET Mode; When the market scale is large, the profits under CT Mode are greater than those under ET Mode. In addition, we compare the profits of the manufacturer in different cases. When the market size is small, the manufacturer have better choose ET Mode and the system has better choose RP case. When the market size is large, the manufacturer have better choose CT Mode and the system has also better choose RP case.

**Fig 13:**
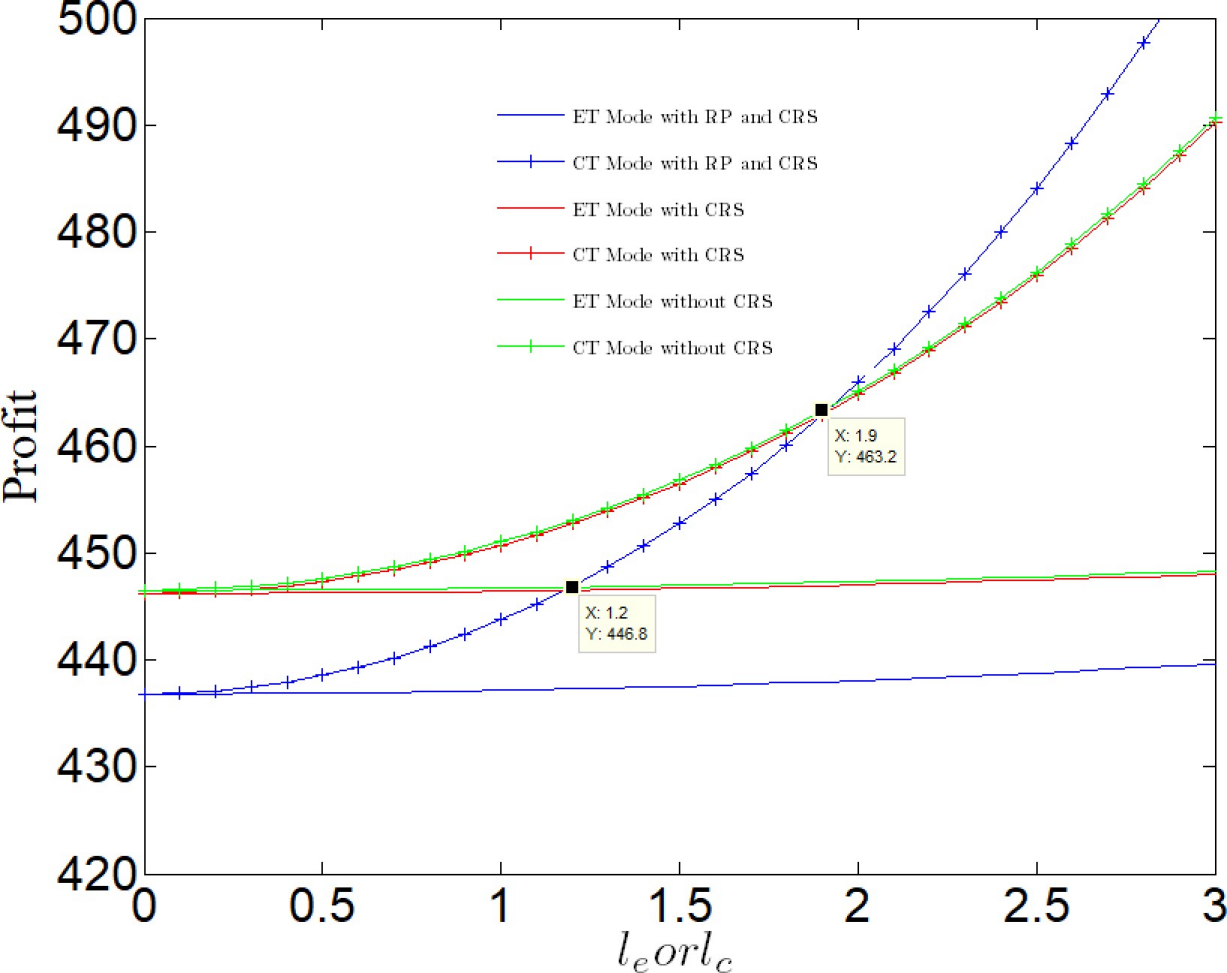
Impact of emission reduction technical efficiency on profit.

Fig 13 analyzes the impact of emission reduction technical efficiency on profit. Regardless of whether CT Mode or ET Mode, the profit increase with the increase of emission reduction technical efficiency. Compared with ET Mode, the profit under CT Mode is more vulnerable to the impact of emission reduction efficiency, and the difference between the two Mode is growing with the increase of emission reduction efficiency. If the emission reduction efficiency *l*_*e*_/*l*_*c*_ <1.2, the profit in RP case is lower than that in case of NN or NR. In the case of PR, if <1.2 < *l*_*e*_/*l*_*c*_ <1.9, profit under CT Mode is greater than that under ET Mode in any case; If the government considers the punishment mechanism, the manufacturer’s profit from adopting clean process technology is greater than that of the manufacturer in any case. When *l*_*e*_/*l*_*c*_ >1.9, if the government considers the punishment mechanism, the profit under CT Mode is greater than that of the manufacturer in any case.

Above, we assume that the emission reduction efficiency of the two technologies is equal, but in the actual production process, due to differences in organizational structure, production process, human resources, etc., the emission reduction efficiency under two modes may not be equal, that is, there is a situation: the emission reduction efficiency of CT Mode is far lower than that of ET Mode. Therefore, when the efficiency of ET Mode is much higher than that of CT Mode, the profit under ET Mode may be greater than that under CT Mode. To sum up, CT Mode is not the best choice.

## 6. Discussion

In order to alleviate environmental pressure, many countries and regions have put forward the goal of carbon neutrality, such as the United States, the European Union, the United Kingdom, etc. As the main body of the ecosystem, manufacturing firms should not only meet the increasingly diverse needs of consumers, but also assume the responsibility of environmental protection. Their decision is related to the realization of product carbon emissions and emission reduction targets. In order to encourage firms to undertake social responsibilities, develop and introduce low-carbon products, governments of all countries have continuously strengthened and improved relevant legislation and exerted influence on firms through various administrative or economic policies to achieve sustainable economic, social and ecological development. Therefore, one of the main objectives of this paper is how the government formulates reward-penalty policies to achieve a win-win situation of social welfare and corporate profits. In the past, most of the literature only considered punitive measures, such as tax collection, or incentive measures, such as subsidies, etc. Few literature combined incentives and punitive measures to study firm emission reduction. As for the research on the government’s reward-penalty policy, most of the literatures take the closed-loop supply chain as the research object to study the impact of the reward-penalty policy on the firm recovery decision and social welfare.

In the actual production process, the manufacturer uses environmental technology to control pollution, mainly including the pollution control technology of pipe ends and cleaner process. One of the main objectives of this study is how manufacturers control pollution to achieve maximum benefits. In other words, what factors affect firms to choose pollution control mode. However, most of the literatures rarely discuss pollution control technologies separately. According to the working principles of clean technology and end-of-pipe pollution control, this paper improves the corresponding models in reference [38] and constructs emission reduction modes under different technology pollution control modes. As far as the author knows, the choice of pollution control mode considering corporate social responsibility and the design of government reward-penalty policies under different pollution control modes have not been discussed at present.

Manufacturing firms are the social main body of pollutant emissions, and taking certain social responsibilities to reduce emissions plays an important role in the sustainable development of economy, society and ecology. Admittedly, the most ideal design and planning of the government is to hope that manufacturing firms take the initiative to assume corporate social responsibility without any support from the government at any policy level. However, through this study, it is found that firms will not be willing to assume corporate social responsibility without external policy incentives, because the goal of firms is to maximize benefits. Therefore, the government should consider the incentive of the policy for firms and the improvement of social welfare when designing reward-penalty policy. As shown in the numerical analysis section, under the CT technology mode, when 0.05 < *β* < 0.26, this policy can achieve the win-win effect of social welfare and corporate profits.

In this paper, market size and emission reduction technology efficiency are the main factors that affect the choice of emission reduction technology mode. However, changing the firm’s emission reduction technology mode is a difficult project, because it will be affected by many factors such as capital, personnel, infrastructure and supporting facilities. In other words, firms will not easily change the emission reduction technology mode unless they encounter new and attractive market opportunities and challenges. Therefore, at the initial stage of investment in new plants, it is necessary to determine which technology emission reduction mode.

## 7. Conclusion

This paper takes a green manufacturer as the research object and considers the demand function affected by consumers’ green preference consciousness. Then, in the absence of government subsidy mechanism, this paper constructs four decision-making models, namely ET model without CSR, ET model with CSR, CT model without CSR, CT model with CSR. The optimal yield and R&D level of the four models are solved respectively. Through analyzing the impact of CSR on social welfare and corporate profits, it is found that corporate social responsibility is conducive to increasing social welfare, but not conducive to improving corporate profits. Therefore, it is necessary for the government to take corresponding measures to improve the enthusiasm of manufacturing firms to reduce emissions for achieving a win-win situation of social welfare and corporate profit. Based on the above analysis, in the case of government subsidy mechanism, considering corporate social responsibility, the models under two emission reduction technology modes are constructed, and the conditions for achieving win-win social welfare and corporate profit are explored through simulation analysis. It also analyzes the impact of other parameters on corporate profits, social welfare and emission reduction mode selection.

The main conclusions of this paper include the following aspects: (1) consumers’ green preference consciousness can improve the profits of firms and is conducive to the development of firms. However, there is a non-linear relationship between consumers’ green preference consciousness and social welfare, that is, there is a threshold value. When consumers’ green preference consciousness is large, consumers’ green preference consciousness can increase social welfare. (2) In the absence of the government’s reward and punishment policies, the corporate social responsibility undertaken by firms is likely to cause a decline in corporate profits, so firms lack the enthusiasm to undertake corporate social responsibility. However, corporate social responsibility can improve social welfare. (3) When a firm undertakes corporate social responsibility, its market demand can increase and its yield can also increase. In other words, the more social responsibility a firm undertakes, the greater the number of production. However, if a firm adopts end-of-pipe pollution control technology, there is no relationship between its technical input and the amount of corporate social responsibility it undertakes. (4) Judging the feasibility of the government’s reward-penalty policy depends on two conditions: one is that the reward-penalty policy can encourage firms to assume corporate social responsibility, and the other is that the reward-penalty policy can improve social welfare. This paper finds that only when the reward-penalty degree of the government is within a certain range, the government will actively implement the reward-penalty policy, and firms can actively assume corporate social responsibility. (5) In this paper, the important factors that affect firms’ choice of pollution control mode are market size and emission reduction efficiency. When the market size is small, firms should adopt end-of-pipe pollution control technology; If the market scale is large, it is beneficial for firms to adopt cleaner process pollution control technology; If the emission reduction efficiency of end-of-pipe pollution control technology is much higher than that of clean process technology, firms should adopt end-of-pipe pollution control technology.

## 8. Managerial implication

Several managerial implications can be derived through our analytical and numerical results.

First, at the stage of project investment planning, firms’ decision-makers should fully investigate the product market size and prospects, and investigate the firms production parameters as accurately as possible. If the market scale is small, firms should adopt end-of-pipe pollution control technology. If the market scale is large, firms should adopt cleaner process pollution control technology. Because under the cleaner process mode, corporate profits are more vulnerable to the impact of market size. In the actual production and operation process, firms should also combine the product development potential and financial constraints, actively tap the existing market space, increase product categories, and try to meet the needs of consumers at different levels.

Second, managers should improve the efficiency of emission reduction technology, because the improvement of emission reduction efficiency is conducive to increasing corporate profits. If conditions permit, firms can improve emission reduction efficiency through the following aspects. (1) Firms should comprehensively analyze existing emission reduction processes, evaluate unreasonable processes, and optimize and restructure them. (2)Firms can train employees online or offline to improve their professional quality, and establish and improve the evaluation system to actively guide their future work behavior and performance. (3) Firms should increase investment in auxiliary infrastructure to ensure efficient operation of emission reduction process.

Third, while encouraging firms to carry out green technology innovation, the government should support firms to adopt reasonable ways to publicize their green production concepts and products, and cultivate consumers’ green preference behavior. The above research conclusion shows that the enhancement of green innovation capability will increase the income of firms, make up for the investment in innovation, improve the competitiveness of firms, and actively promote the development of green technology innovation. This transmission process will form a positive reinforcement ring. In addition, the government should support firms to publicize their green production concepts and products through corresponding platforms to improve their social reputation, enhance their product market recognition, cultivate consumers’ green preference and increase the demand for products. The government should also create a supportive atmosphere for green production in the whole society, so as to stimulate consumers’ enthusiasm for supporting green products and consolidate the consumption base on the demand side of products. In short, it is necessary to make green products more valuable and value-added.

Last but not the least, since the imbalance of regional development exists in all economies, local governments should formulate reasonable and feasible reward-penalty policy based on development practices to promote low-carbon emission reduction. From the above analysis, it can be found that corporate social responsibility is conducive to social welfare and not conducive to corporate profits, while the reward-penalty policy is conducive to increasing corporate profits, but only to a certain extent can improve social welfare. Therefore, local governments need to fully investigate the local economic development, weigh social welfare and economic development, and formulate reasonable reward-penalty policy in combination with the financial budget. A reasonable reward-penalty policy should meet the following two conditions: one is improving the enthusiasm of firms to undertake social responsibilities, and the other is stimulating the initiative of the government to formulate reward-penalty policy.

The proposed model has some limitations. (1)The paper takes a manufacturer’s emission reduction as the research object, and does not involve other members of the supply chain. However, in practice, the entire supply chain has shared the economic benefits of emission reduction. In the future, the downstream retailers can be considered to share the cost of emission reduction, and the relationship between the cost sharing rate and the selection of emission reduction technologies can be discussed. (2)We study the reward-penalty policy, while the government has a variety of environmental regulations. In the future, we can compare the difference between emission reduction cost subsidies and emission reduction effect subsidies. (3) We do not consider the financial constraints of manufacturers. In the future, we can consider how to select emission reduction technologies under the financial constraints of manufacturers.
